# Elevated high-density lipoprotein triglycerides increase atherosclerotic risk

**DOI:** 10.1016/j.jlr.2025.100791

**Published:** 2025-03-29

**Authors:** Weifang Liu, Shaoze Chen, Chengzhang Yang, Fang Lei, Xuewei Huang, Xingyuan Zhang, Tao Sun, Lijin Lin, Chuansen Wang, Yuanyuan Cao, Zhi-Gang She, Xuan Xiao, Hongliang Li

**Affiliations:** 1 Department of Cardiology, Renmin Hospital of Wuhan University, Wuhan, Hubei Province, China; 2 State Key Laboratory of New Drug Discovery and Development for Major Diseases, Gannan Medical University, Ganzhou, China; 3 Gannan Innovation and Translational Medicine Research Institute, Gannan Medical University, Ganzhou, China; 4 Department of Cardiology, Huanggang Central Hospital of Yangtze University, Huanggang, China; 5 Medical Science Research Center, Zhongnan Hospital of Wuhan University, Wuhan, China; 6 Department of Cardiology, The Third Xiangya Hospital, Central South University, Changsha, China; 7 School of Basic Medical Sciences, Wuhan University, Wuhan, China; 8 Department of Ophthalmology, Renmin Hospital of Wuhan University, Wuhan, Hubei, China

**Keywords:** coronary artery disease, high-density lipoprotein, mendelian randomization, omega-3 fatty acids, triglycerides in high-density lipoprotein

## Abstract

The relationship between high-density lipoprotein (HDL) and atherosclerotic risk remains incompletely elucidated, potentially due to the inherent heterogeneity of HDL particles. Hypertriglyceridemia is associated with alterations in HDL composition. This study investigated the impact of elevated triglycerides (TG) on HDL and its association with coronary artery disease (CAD) risk using a large prospective cohort study and Mendelian randomization (MR). We found that elevated TG was associated with reduced HDL particle size, decreased concentrations of HDL components, and increased triglycerides in HDL (HDL-TG) (all *P* for trend < 0.001). The protective effects of HDL particle concentration and HDL cholesterol on CAD are attenuated with increasing serum TG levels. An independent and positive association between HDL-TG levels and incident CAD events (hazard ratio [HR] per 1 standard deviation increase: 1.066, 95% CI: 1.052–1.080, *P* < 0.001) was confirmed even after adjustment for established cardiovascular disease risk factors. MR analyses supported a causal role for HDL-TG in CAD development (inverse-variance weighted [IVW] method: odds ratios [ORs] of 1.120 (95% CI: 1.053–1.192, *P* < 0.001) and 1.141 (95% CI: 1.032–1.263, *P* = 0.010) for dataset groups 1 and 2, respectively). Drug-target MR analyses suggested a potential association between omega-3 fatty acids (OM3-FA) and lower HDL-TG levels, with *LPL* and *DGAT2* as key pharmacological targets. Our findings suggest that elevated TG contributes to adverse alterations in HDL, elevating CAD risk. HDL-TG is an independent positive risk factor for CAD and a potential causal contributor to CAD development. OM3-FA supplementation may offer a therapeutic strategy for mitigating the CAD risk associated with elevated HDL-TG.

Atherosclerosis is the primary cause of atherosclerotic cardiovascular disease (ASCVD), including coronary artery disease (CAD), stroke, and peripheral artery disease as well as conditions such as retinal artery occlusion and other vascular diseases. Among these detrimental diseases, ASCVD remains the leading cause of morbidity and mortality worldwide, posing a significant public health burden (1, 2, 3, 4). High-density lipoprotein (HDL) plays multifaceted roles in lipid metabolism and atherosclerosis (5, 6, 7), but its association with ASCVD is more complex than previously recognized (6, 7, 8, 9). Although epidemiological studies generally demonstrate an inverse relationship between HDL cholesterol (HDL-C) levels and ASCVD risk, clinical trials aimed at increasing HDL-C levels have not consistently shown a reduction in ASCVD events (5, 6, 8, 10, 11, 12, 13). Mendelian randomization (MR) studies have also provided inconclusive evidence for the causal protective effect of HDL-C on ASCVD (14, 15). This inconsistency likely stems from the heterogeneity of HDL particles, with certain subfractions potentially exhibiting distinct properties (6, 7, 8, 9). Therefore, relying solely on HDL-C levels may be insufficient to assess the full impact of HDL on ASCVD risk.

Elevated triglyceride (TG) levels are associated with an increased risk of ASCVD (16, 17, 18). Studies have shown that elevated TG can induce structural and functional alterations in HDL particles, including decreased particle diameter, altered composition (increased TG in HDL [HDL-TG] levels), and impaired anti-inflammatory and antioxidant properties, which may compromise HDL's anti-atherosclerotic capacity (19, 20, 21). However, there is a lack of epidemiological evidence linking TG-induced HDL compositional changes with ASCVD risk, and evidence regarding the association between HDL-TG levels and ASCVD risk is limited. Currently, only one small-scale cross-sectional study from Spain has investigated the relationship between HDL-TG levels and subclinical atherosclerotic events in patients with metabolic syndrome and type 2 diabetes (22). The small sample size and study design limitations restrict the generalizability of its conclusions. Other sparse evidence primarily comes from studies focusing on the impact of overall metabolomic lipid profiles on ASCVD risk, where the role of HDL-TG is largely overlooked compared to other well-established lipid markers (23, 24, 25, 26). Moreover, these studies did not adequately account for other confounding factors, such as other lipids, inflammation, HDL particle concentration (HDL-P), and size, to address whether HDL-TG is an independent and causal risk factor for ASCVD. Furthermore, there is currently a lack of research exploring potential therapeutic targets and interventions for HDL-TG, which may be crucial for mitigating ASCVD risk arising from changes in HDL composition.

Therefore, this study, encompassing an analysis of a large prospective cohort and MR studies, aims to comprehensively investigate the relationships among elevated serum TG, alterations in HDL size and composition, and their potential association with CAD risk, with a particular focus on HDL-TG. This study may enhance our understanding of the role of HDL in the pathogenesis of CAD and provide potential new strategies for mitigating CAD risk associated with altered HDL characteristics.

## Materials and Methods

### Study design and data sources

Our study integrated data from both a prospective cohort study and MR analysis. For the observational analysis, data were derived from the UK Biobank, which recruited approximately 500,000 participants aged 40–69 at the time of recruitment in 2006–2010 (27). All participants provided written informed consent, and the North West Multicentre Ethics Committee granted ethical approval to UK Biobank. All human studies were conducted in accordance with the Declaration of Helsinki. This study was performed under UK Biobank application number 77195. Among all 502,412 participants in the UK Biobank, we excluded participants who withdrew consent at the time of the study (n = 43), who had no information on HDL-related metabolomics items (n = 228,017), and who had CAD at baseline (n = 15,186), leaving 259,166 participants for final analysis (Supplemental Fig. 1).

This two-sample MR study was based on publicly available summary datasets, with detailed information provided in Supplemental Table 1A (28, 29, 30). The drug-target MR analysis utilized cis-eQTL summary statistics obtained from the eQTLGen Consortium (additional details are provided in Supplemental Table 1B) (31).

The Supplementary Methodos section 1 describes in detail the study design and data sources.

### Observational study

#### Classification of total TG

To assess the changes in HDL size and composition and its association with CAD with variations in TG levels, we classified the clinically routine measurements of TG into five groups based on the ATP III 4 TG categories and the fourth Adult Treatment Panel guidelines (low-normal to severe hypertriglyceridemia) (details are provided in the Supplementary Methods section 2) (32, 33).

#### Measurement of NMR-based HDL-related metabolomics

Metabolic biomarkers were assessed using a high-throughput nuclear magnetic resonance (NMR)-based metabolic biomarker profiling platform developed by Nightingale Health Ltd (Supplementary Methods section 2). We utilized data on HDL-related metabolites, including the average diameter of HDL particles, HDL-P, total lipids in HDL, HDL-C, HDL-TG, phospholipids in HDL, cholesteryl esters in HDL, and free cholesterol in HDL. Additionally, detailed lipid subfractions contained within four size categories of lipoprotein particles, ranging from small HDL-P to very large HDL-P, were analyzed. Furthermore, our study also incorporated additional metabolite data, including total TG, total cholesterol, low-density lipoprotein cholesterol (LDL-C), and apolipoprotein B (ApoB). Prior to statistical analysis, all metabolite concentration values were scaled to unit variance (mean = 0, standard deviation (SD) = 1) to ensure comparability across samples and to mitigate potential batch effects.

#### Ascertainment of CAD

The primary outcome of this study was the occurrence of CAD (International Classification of Diseases (ICD10): I20-I25) (Supplemental Table 2 and Supplementary Methods section 2). Follow-up commenced upon participant enrollment and continued until the first diagnosis of CAD, loss of follow-up, death, or the conclusion of the study (November 28, 2022), whichever came first.

#### Calculation of the general cardiovascular risk score

To investigate whether the independent association between HDL-TG and incident CAD events is influenced by participants' general cardiovascular risk levels, we calculated a general cardiovascular risk score for study participants using the Framingham risk score (FRS) model (details are provided in the Supplementary Methods section 2) (34, 35). Participants were then categorized into "low cardiovascular diseases (CVD) risk" and "high CVD risk" groups based on the median FRS in the population (36).

#### Covariate measurement

We considered potential confounders based on various data sources, including touchscreen questionnaires, physical measurements, biochemical indices, and medical history. These variables encompassed age, sex (female/male), systolic blood pressure (SBP), diastolic blood pressure (DBP), body mass index (BMI), blood glucose, C-reactive protein (CRP), UK Biobank assessment center, overall health rating (excellent; good; fair; poor), Townsend deprivation index (an area-based proxy measure for socioeconomic status considering household overcrowding, car ownership, owner occupation, and unemployment, with a higher index indicating poorer socioeconomic status) (37), education qualifications (college or university degree; A/AS levels, NVQ, HND, HNC, other professional qualifications, and equivalent; O levels/GCSEs or CSEs or equivalent; none of the above), smoking status (never; previous; current), alcohol drinker status (never; previous; current), ethnicity (White, Asian or Asian British, Black or Black British, Mixed, Chinese, or other ethnic group), baseline hypertension, baseline diabetes, baseline stroke, and baseline medication use (including cholesterol-lowering drugs, antihypertensive medications, insulin, etc.). Variables and codes used for confounder assessment are provided in Supplemental Table 3.

### Two-sample MR analysis

To investigate the potential causal association between HDL-TG and CAD, we performed two-sample MR analyses. Genetic instruments were selected based on genome-wide significance (*P* < 5 × 10^-8^), linkage disequilibrium (LD) clumping (*R*^*2*^ < 0.1 within a 10,000 kB window), and *F* statistics ≥ 10 (38, 39, 40). All single-nucleotide polymorphisms (SNPs) used as instrumental variables are listed in Supplemental Table 4. Univariable MR methods (inverse variance weighted [IVW] (41), weighted median (42), MR-Egger (43), weighted mode, MR-Egger intercept (41), Cochrane’s Q statistic (43), MR pleiotropy residual sum and outlier [MR-PRESSO]) (44) as well as multivariable MR((45)) (adjusting for HDL-C, LDL-C, and total TG) were applied (see Supplementary Methods, Section 3).

### Drug-target MR

To explore the potential impact of existing lipid-lowering medications on HDL-TG indicators, we conducted further drug-target MR studies. Initially, we conducted a comprehensive search within the DrugBank database (https://go.drugbank.com/) to identify medications associated with treating hypertriglyceridemia and lowering LDL-C levels. Our focus was on genes encoding the pharmacological targets of fenofibric acid, omega-3 fatty acids (OM3-FA), and statins (46). Leveraging the availability of cis-eQTL data, our final analysis included the following genetic loci: peroxisome proliferator-activated receptor alpha (*PPARA*) (the primary pharmacological target for fenofibric acid), diacylglycerol o-acyltransferase 2 (*DGAT2*), lipoprotein lipase (*LPL*), elongation of very long-chain fatty acids protein 4 (*ELOVL4*) (the primary pharmacological targets for OM3-FA), and 3-hydroxy-3-methylglutaryl-CoA reductase (*HMGCR*) (the primary pharmacological target for statins). Furthermore, given that cholesteryl ester transfer protein (*CETP*) allows the net movement of cholesteryl ester from HDL to TG-rich very low-density lipoprotein (VLDL), and the equimolar transport of TG from VLDL to HDL (47), and despite the lack of approved *CETP*-targeting drugs, we included *CETP* in our analysis to explore its potential impact on HDL-TG levels. Detailed information about the target genes is provided in Supplemental Table 1C. Subsequently, we selected SNPs located within the upstream or downstream 100 kb of the identified targets, achieving genome-wide significance (*P* < 5 × 10^-8^), and clumped them for an LD threshold of *R*^*2*^ < 0.1. We identified three variants in the *PPARA* locus, four variants in the *DGAT2* locus, 18 variants in the *LPL* locus, 11 variants in the *ELOVL4* locus, 3 variants in the *HMGCR* locus, and 6 variants in the *CETP* locus (Supplemental Table 5). We employed the same analytical methods as in the univariable MR analysis.

### Additional analyses

Supplementary analyses were performed to (a) evaluate the associations and potential causal effects of VLDL and low-density lipoprotein (LDL) subcomponents (including LDL-TG and VLDL-TG) with CAD, (b) investigate the relationship between the HDL-TG/HDL-C ratio and incident CAD, and (c) assess the causal role of HDL-C in CAD. Detailed methods for these analyses are provided in the Supplementary Methods, Section 4.

### Statistical analysis

#### Observational study

Continuous variables were expressed as median and interquartile range (IQR) values. Categorical variables were expressed as frequencies and percentages. Comparisons of the differences between groups were performed with the Mann-Whitney U test for non-normally distributed continuous variables and the χ^2^ test or Fisher’s exact test for categorical variables. Trend *P*-values for differences between groups were calculated using the 'compareGroups' package in R. The missing data on the variables were imputed using the chained equations method implemented in the MICE package in R. The correlation between total TG and HDL-related metabolites was assessed using the Spearman correlation method. The association between the HDL-related metabolites and the incidence of CAD during the follow-up period was analyzed using the Cox proportional hazard regression model. The Cox models were adjusted for age, sex, ethnic background, overall health rating, education qualifications, smoking status, alcohol drinker status, assessment center, Townsend deprivation index, history of diabetes, history of hypertension, lipid-lowering therapy, antihypertensive therapy, insulin therapy, SBP, glucose, and BMI. The model additionally adjusted for total TG levels when evaluating the relationship between HDL-P, HDL-C, and incident CAD events across different TG levels. The hazard ratio (HR) and 95% confidence intervals (CI) were reported. A 2-sided *P* value < 0.05 was considered statistically significant. In addition, we employed restricted cubic spline (RCS) models fitted with Cox regression to assess the potential non-linear relationship between HDL-TG on a continuous scale and CAD. We chose three knots, and nonlinear tests were conducted using analysis of variance.

#### Two-sample MR analysis

Results were reported as the odds ratio (OR) with 95% CI for univariable and multivariable MR analysis and β with standard errors (SE) for drug-target MR. Two-sample MR analyses were performed using the TwoSampleMR and MRPRESSO packages.

All analyses were performed using R software version 4.2.2 (R Foundation for Statistical Computing).

## Results

### Baseline characteristics of participants stratified by incident CAD

Table 1 presents the baseline characteristics of 259,166 participants from the prospective cohort study, categorized by incident CAD during follow-up. The median age of overall participants at recruitment was 57.0 years, and 44.7% of participants were male. Participants who developed CAD were older by 7.0%, had 5.2% higher SBP, 2.4% higher DBP, 4.9% higher BMI, 1.6% higher blood glucose levels, and 1.4 times higher CRP levels compared to those who did not develop CAD. Regarding lifestyle factors, socio-economic factors, and comorbidities, the CAD group reported poorer overall health, lower education qualifications, and had approximately 1.5 times higher prevalence of current smoking and previous alcohol drinking. Additionally, the CAD group had a 14.2% higher median Townsend deprivation index, a 2.8 times higher prevalence of previous stroke, a 1.8 times higher prevalence of previous hypertension, and a 2.7 times higher prevalence of diabetes. Consequently, they were more frequently prescribed cholesterol-lowering drugs (2.1 times), antihypertensive drugs (2.0 times), and insulin (3.5 times) (all *P* < 0.001).

**Table 1:** Baseline characteristics of the longitudinal participants categorized by CAD incidence

Characteristics	Overall	Non-CAD	CAD	*P*-value
	(N = 259,166)	(N = 237,312)	(N = 21,854)	
Biological and clinical characteristics of subjects				
Age at recruitment (year, median(IQR))	57 [50, 63]	57 [49, 63]	61 [56, 65]	<0.001
Sex, n (%)				
Male	115,846 (44.7)	102,311 (43.1)	13,535 (61.9)	<0.001
Female	143,320 (55.3)	135,001 (56.9)	8,319 (38.1)	<0.001
SBP (mmHg, median(IQR))	136.0 [124.5, 149.5]	135.5 [124.0, 148.5]	142.5 [131.0, 155.5]	<0.001
DBP (mmHg, median(IQR))	82.0 [75.5, 89.0]	82.0 [75.0, 88.5]	84.0 [77.0, 91.0]	<0.001
BMI (kg/m^2^, median(IQR))	26.7 [24.1, 29.8]	26.6 [24.0, 29.6]	27.9 [25.2, 31.2]	<0.001
Glucose (mmol/L, median(IQR))	4.92 [4.59, 5.30]	4.92 [4.59, 5.29]	5.00 [4.64, 5.45]	<0.001
CRP (mg/L, median(IQR))	1.32 [0.65, 2.74]	1.29 [0.64, 2.67]	1.77 [0.91, 3.56]	<0.001
Overall health rating, n (%)				<0.001
Excellent	43,894 (17.0)	41,730 (17.7)	2,164 (10.0)	
Good	152,489 (59.2)	141,171 (59.8)	11,318 (52.2)	
Fair	51,597 (20.0)	45,212 (19.2)	6,385 (29.5)	
Poor	9,742 (3.8)	7,944 (3.4)	1798 (8.3)	
Education qualifications, n (%)				<0.001
College or university degree	83,817 (32.7)	78,488 (33.4)	5,329 (24.8)	
A/AS levels, NVQ, HND, HNC, other professional qualifications, and equivalent	59,222 (23.1)	54,200 (23.1)	5,022 (23.4)	
O levels/GCSEs or CSEs or equivalent	70,145 (27.4)	64,672 (27.6)	5,473 (25.4)	
None of the above	43,052 (16.8)	37,365 (15.9)	5,687 (26.4)	
Smoking status, n (%)				<0.001
Never	143,424 (55.6)	133,499 (56.5)	9,925 (45.7)	
Previous	87,531 (33.9)	78,979 (33.4)	8,552 (39.4)	
Current	26,995 (10.5)	23,759 (10.1)	3,236 (14.9)	
Alcohol drinker status, n (%)				<0.001
Never	10,963 (4.2)	9,849 (4.2)	1,114 (5.1)	
Previous	8,828 (3.4)	7,726 (3.3)	1,102 (5.1)	
Current	238,776 (92.4)	219,212 (92.6)	19,564 (89.8)	
Ethnic background, n (%)				<0.001
White	245,394 (95.1)	224,754 (95.1)	20,640 (94.9)	
Mixed	1,458 (0.6)	1,350 (0.6)	108 (0.5)	
Asian or Asian British	4,469 (1.7)	3,877 (1.6)	592 (2.7)	
Black or Black British	3,719 (1.4)	3,506 (1.5)	213 (1.0)	
Chinese	788 (0.3)	756 (0.3)	32 (0.2)	
Other ethnic group	2,196 (0.9)	2039 (0.9)	157 (0.7)	
Townsend deprivation index, median(IQR)	−2.23 [−3.69, 0.37]	−2.26 [−3.70, 0.32]	−1.94 [−3.53, 0.97]	<0.001
Comorbidity/medication history, n (%)				
Stroke	3,344 (1.3)	2,676 (1.1)	668 (3.1)	<0.001
Hypertension	66,256 (25.6)	57,057 (24.0)	9,199 (42.1)	<0.001
Diabetes	11,829 (4.6)	9,468 (4.0)	2,361 (10.8)	<0.001
Cholesterol-lowering drug	36,706 (14.2)	30,766 (13.0)	5,940 (27.2)	<0.001
Antihypertensive drug	47,546 (18.4)	40,227 (17.0)	7,319 (33.5)	<0.001
Insulin	2,400 (0.9)	1792 (0.8)	608 (2.8)	<0.001
Follow up time (year, median(IQR))	13.7 [12.8, 14.4]	13.8 [13.1, 14.5]	7.5 [4.2, 10.7]	<0.001
Concentrations of plasma lipids and HDL components				
Clinical-Triglycerides^a^	1,475.0 [1,042.0,2136.0]	1,452.0 [1,029.0,2103.0]	1740.0 [1,225.3,2487.0]	<0.001
Total Triglycerides	1,202.6 [878.6,1640.1]	1,188.8 [869.5,1622.5]	1,363.9 [997.3,1814.4]	<0.001
Total Cholesterol	4,644.6 [4,054.1,5265.9]	4,651.9 [4,067.4,5269.5]	4,553.5 [3,899.1,5225.7]	<0.001
LDL Cholesterol	1741.8 [1,467.0,2040.3]	1742.1 [1,469.9,2038.8]	1738.1 [1,431.5,2057.6]	0.001
Apolipoprotein B	842.7 [717.1,981.5]	841.7 [717.4,979.4]	853.4 [713.2,1004.2]	<0.001
Concentration of HDL Particles	15.2 [13.7,16.9]	15.3 [13.7,16.9]	14.7 [13.2,16.3]	<0.001
Average Diameter for HDL Particles (nm)	9.6 [9.5, 9.8]	9.6 [9.5, 9.8]	9.6 [9.5, 9.7]	<0.001
Total Lipids in HDL	2,972.9 [2,583.2,3432.5]	2,989.6 [2,597.7,3449.4]	2,798.6 [2,450.0,3227.7]	<0.001
HDL Cholesterol	1,288.8 [1,093.9,1530.7]	1,298.9 [1,102.2,1541.0]	1,183.0 [1,020.1,1402.9]	<0.001
Triglycerides in HDL	138.7 [111.6,171.4]	137.9 [111.0,170.5]	147.0 [118.8,181.5]	<0.001
Cholesteryl Esters in HDL	1,004.1 [848.4,1194.0]	1,012.2 [855.4,1202.4]	918.4 [788.3,1091.1]	<0.001
Phospholipids in HDL	1,535.0 [1,338.2,1761.6]	1,542.5 [1,345.2,1769.2]	1,453.9 [1,275.3,1669.9]	<0.001
Free Cholesterol in HDL	286.2 [244.0,338.4]	287.9 [245.5,340.6]	266.9 [230.4,313.9]	<0.001
Total Lipids in Very Large HDL	149.9 [113.4,204.6]	151.7 [114.5,207.5]	134.0 [103.3,174.0]	<0.001
Phospholipids in Very Large HDL	68.4 [47.8,99.5]	69.4 [48.4,101.2]	59.4 [42.5,82.0]	<0.001
Cholesterol in Very Large HDL	74.5 [58.4,98.3]	75.3 [59.0,99.6]	67.3 [53.6,84.7]	<0.001
Cholesteryl Esters in Very Large HDL	52.5 [39.5,72.0]	53.2 [40.0,73.1]	46.3 [35.6,60.4]	<0.001
Free Cholesterol in Very Large HDL	22.2 [18.6,26.7]	22.3 [18.7,26.9]	21.1 [17.7,24.7]	<0.001
Triglycerides in Very Large HDL	6.8 [5.3,8.6]	6.7 [5.3,8.6]	7.0 [5.5,9.0]	<0.001
Total Lipids in Large HDL	587.7 [416.3,838.6]	597.7 [422.7,850.5]	494.9 [362.9,692.5]	<0.001
Phospholipids in Large HDL	297.2 [213.8,414.5]	301.8 [216.9,419.7]	253.7 [189.2,349.0]	<0.001
Cholesterol in Large HDL	261.7 [173.7,393.1]	267.2 [177.2,399.7]	210.4 [145.8,312.4]	<0.001
Cholesteryl Esters in Large HDL	202.4 [133.1,304.9]	206.7 [135.8,310.2]	161.4 [110.9,241.3]	<0.001
Free Cholesterol in Large HDL	59.6 [40.4,88.4]	60.7 [41.1,89.8]	49.6 [34.5,71.4]	<0.001
Triglycerides in Large HDL	28.7 [21.9,37.2]	28.7 [21.9,37.2]	28.8 [21.8,37.3]	0.351
Total Lipids in Medium HDL	1,032.4 [889.8,1189.0]	1,037.2 [894.7,1193.3]	978.2 [843.1,1135.5]	<0.001
Phospholipids in Medium HDL	485.5 [422.2,554.4]	487.5 [424.1,556.0]	464.5 [402.9,534.7]	<0.001
Cholesterol in Medium HDL	491.6 [413.5,579.8]	494.9 [416.6,582.8]	455.3 [384.4,540.7]	<0.001
Cholesteryl Esters in Medium HDL	405.3 [342.3,476.3]	408.0 [345.0,478.6]	375.6 [318.0,444.6]	<0.001
Free Cholesterol in Medium HDL	86.3 [71.0,103.9]	86.9 [71.5,104.5]	80.0 [65.8,96.8]	<0.001
Triglycerides in Medium HDL	52.1 [41.0,65.2]	51.8 [40.7,64.9]	55.1 [43.7,68.7]	<0.001
Total Lipids in Small HDL	1,162.7 [1,063.8,1269.4]	1,162.7 [1,063.9,1269.4]	1,163.3 [1,063.0,1269.9]	0.853
Phospholipids in Small HDL	662.3 [604.9,725.6]	662.3 [605.1,725.6]	661.3 [603.1,726.0]	0.127
Cholesterol in Small HDL	447.1 [408.5,488.5]	447.5 [408.9,488.9]	443.1 [404.2,485.0]	<0.001
Cholesteryl Esters in Small HDL	331.6 [301.6,363.5]	331.9 [301.9,363.7]	328.0 [298.1,360.0]	<0.001
Free Cholesterol in Small HDL	115.6 [105.4,126.6]	115.6 [105.5,126.6]	115.1 [104.7,126.3]	<0.001
Triglycerides in Small HDL	51.4 [40.7,63.5]	50.9 [40.3,63.0]	56.3 [45.7,68.4]	<0.001

BMI, body mass index; CAD, coronary artery disease; CRP, C-reactive protein; DBP, diastolic blood pressure; HDL, high density lipoprotein; IQR, interquartile range; LDL, low-density lipoprotein; SBP, systolic blood pressure.

a Measured by standard clinical chemistry assays.

Analysis of lipid profiles revealed significant differences between the two groups (Table 1). Specifically, the CAD group had a 14.7% higher median level of total TG, 1.4% higher ApoB, 2.1% lower total cholesterol, and 0.2% lower LDL-C. In terms of HDL characteristics, the CAD group exhibited a 3.9% smaller median HDL-P, 6.4% lower total lipids in HDL, 8.9% lower HDL-C, 9.3% lower cholesteryl esters, 5.7% lower phospholipids, and 7.3% lower free cholesterol in HDL. Additionally, the CAD group had a 6.6% higher HDL-TG compared to the non-CAD group. Further investigation of HDL subcomponents demonstrated a consistent pattern of lower levels of cholesteryl esters, phospholipids, and free cholesterol in the CAD group across various HDL subclasses, particularly within the very large and large HDL fractions, while showing higher levels of TG, specifically within smaller HDL particles.

### Elevated serum TG levels are associated with alterations in HDL size and composition, potentially modifying the association between HDL and CAD

To examine the impact of elevated serum TG on HDL characteristics and their potential association with CAD, we performed descriptive and COX regression analyses stratified by serum TG levels to assess changes in HDL particle diameter and subfraction composition, as well as the relationship between HDL-P and HDL-C levels and incident CAD events.

**Fig. 1:**
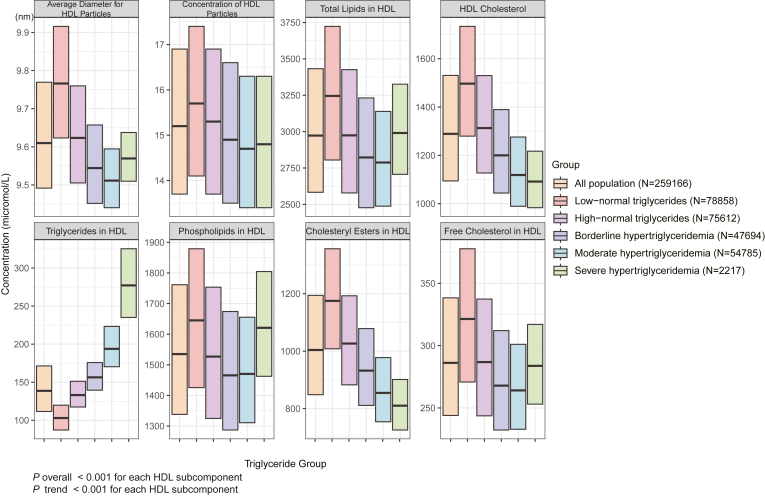
Baseline characteristics of HDL subcomponents by grouping of triglyceride levels. HDL, high-density lipoprotein.

Figure 1 presents the baseline characteristics of HDL subcomponents stratified by TG levels. Apart from the severe hypertriglyceridemia group (N = 2,217), a progressive decrease in average HDL particle diameter was observed with increasing TG levels (*P* for trend <0.001), ranging from 9.77 nm in the low-normal TG group to 9.51 nm in the moderate hypertriglyceridemia group. This trend was accompanied by a significant reduction in HDL-P (*P* for trend <0.001) across increasing TG categories. Furthermore, the concentrations of major HDL components, including HDL-C, phospholipids, and cholesteryl esters, demonstrated a significant negative association with rising TG levels (all *P* for trend <0.001). Conversely, the content of HDL-TG exhibited a significant positive association with increasing TG levels (*P* for trend <0.001). Supplemental Table 6 provides further evidence through correlation analysis, demonstrating that total TG levels were inversely correlated with average HDL particle diameter, HDL-C, phospholipids, and cholesteryl esters concentrations (all *P* < 0.001). In contrast, a strong positive correlation was observed between total TG and HDL-TG content (r = 0.835, *P* < 0.001).

**Fig. 2:**
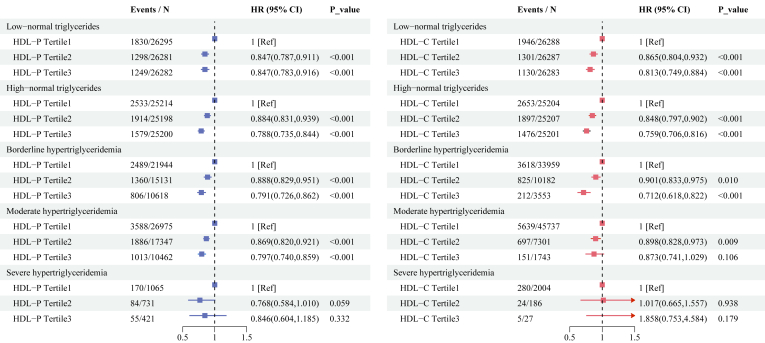
Association between HDL-P or HDL-C and incident coronary artery disease events across triglyceride levels. Model adjusted for age, sex, ethnic background, overall health rating, education qualifications, smoking status, alcohol drinker status, assessment centre, Townsend deprivation index, history of diabetes, history of hypertension, lipid-lowering therapy, antihypertensive therapy, insulin therapy, systolic blood pressure, glucose, body mass index, and total triglyceride. CI, confidence intervals; HDL, high density lipoprotein; HDL-C, HDL Cholesterol; HDL-P, Concentration of HDL Particles; HR, hazard ratio.

Figure 2 and Supplemental Tables 7 and 8 illustrate the association between HDL-P and HDL-C and incident CAD events across various TG levels. In the lower TG groups (low-normal, high-normal, and borderline hypertriglyceridemia), both higher HDL-P and HDL-C levels were significantly associated with a reduced risk of CAD events (all *P* < 0.001). However, as TG levels increased further, the protective effect of HDL appeared to diminish gradually. In the moderate hypertriglyceridemia group, the association between both HDL-P and HDL-C with CAD risk was attenuated, with HR closer to 1 and wider CI (HDL-P, HR: 0.797, 95% CI: 0.740–0.859, *P* < 0.001; HDL-C, HR: 0.873, 95% CI: 0.741–1.029, *P* = 0.106; after fully adjusted confounders). In the severe hypertriglyceridemia group, due to the limited sample size, the results were inconclusive, and no definitive conclusions could be drawn regarding the association between HDL and CAD risk.

### Differential impact of HDL components on CAD risk across HDL particle sizes

**Fig. 3:**
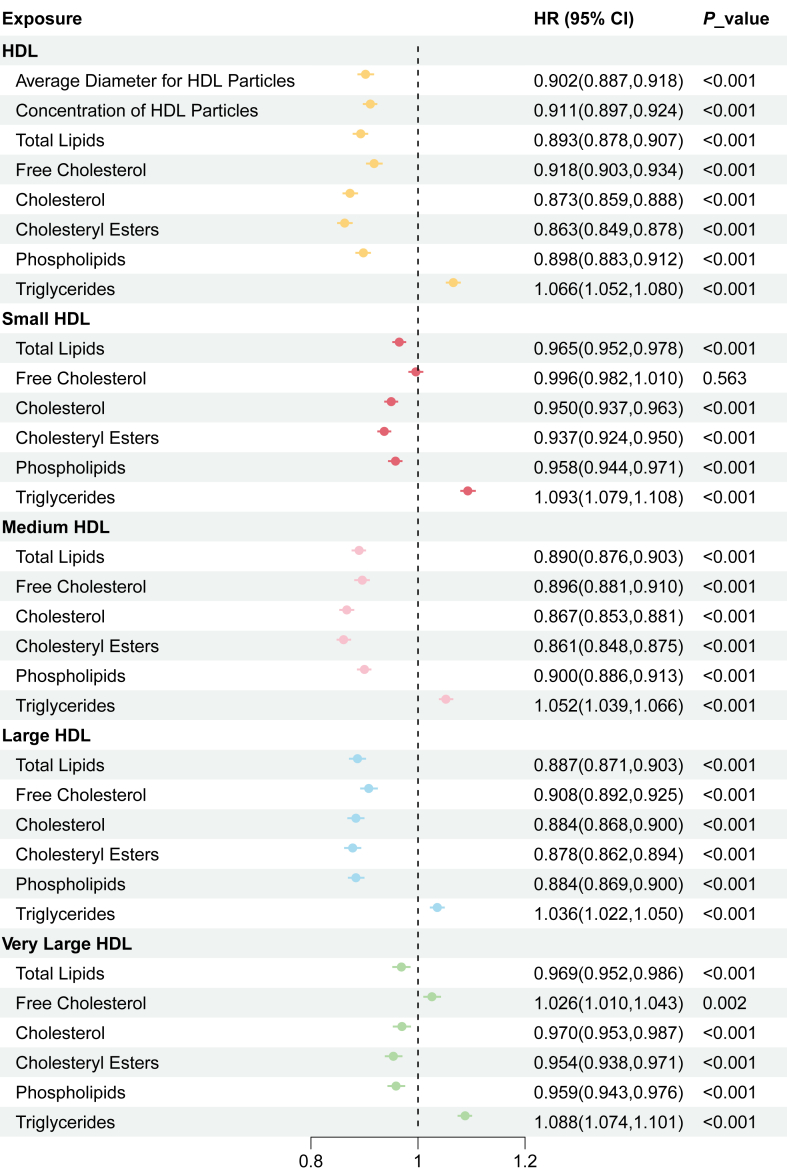
Association of subcomponents in HDL with incident coronary artery disease events across different levels of HDL particle diameter. Model adjusted for age, sex, ethnic background, overall health rating, education qualifications, smoking status, alcohol drinker status, assessment Center, Townsend deprivation index, history of diabetes, history of hypertension, lipid-lowering therapy, antihypertensive therapy, insulin therapy, systolic blood pressure, glucose, and body mass index. CI, confidence intervals; HDL, high density lipoprotein; HR, hazard ratio.

To further investigate the differential influence of HDL size and composition on CAD risk, we examined the relationship between HDL subfraction components and CAD events across different HDL particle diameters. After fully adjusting for potential confounders, HDL-C, phospholipids, cholesteryl esters, free cholesterol, total lipids, HDL-P, and average HDL particle diameter were all significantly associated with a lower risk of CAD events (all *P* < 0.001). However, HDL-TG content exhibited a significant positive association with CAD events (HR: 1.066, 95% CI: 1.052–1.080, *P* < 0.001) (Fig. 3).

When further exploring the association between HDL subcomponents and CAD events across different levels of HDL particle diameter, we found that compared to large and medium HDL particles, components within small and very large HDL particles, including HDL-C, phospholipids, cholesteryl esters, free cholesterol, and total lipids, demonstrated diminished protective associations with CAD, while the detrimental effect of HDL-TG became more pronounced. Specifically, HDL-TG content in the smallest HDL particle size was significantly and positively associated with CAD risk (HR: 1.093, 95% CI: 1.079–1.108, *P* < 0.001), with a similarly strong association observed in very large HDL particles (HR: 1.088, 95% CI: 1.074–1.101, *P* < 0.001) (Fig. 3).

### Robust positive association of HDL-TG with incident CAD: observational evidence

**Fig. 4:**
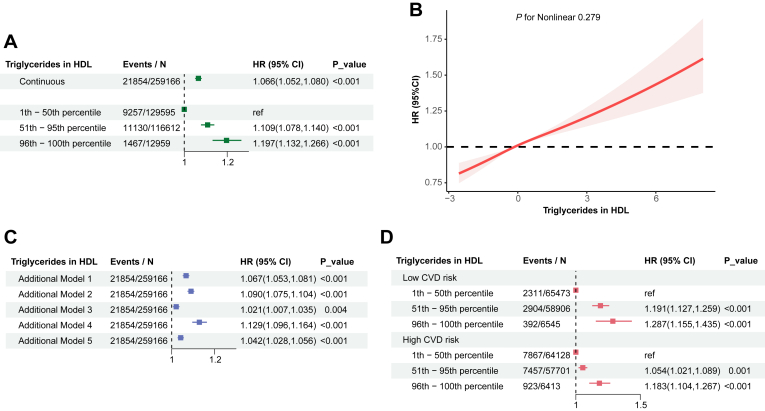
Association of HDL-TG with incident CAD events in observational studies. A, B and D: model adjusted for age, sex, ethnic background, overall health rating, education qualifications, smoking status, alcohol drinker status, assessment centre, Townsend deprivation index, history of diabetes, history of hypertension, lipid-lowering therapy, antihypertensive therapy, insulin therapy, systolic blood pressure, glucose, and body mass index. C: Additional Model 1 was adjusted as in the main model, with additional adjustment for C-reactive protein. Additional Model 2 was adjusted as in the main model, with additional adjustment for concentration of HDL particles and average diameter for HDL particles. Additional Model 3 was adjusted as in the main model, with additional adjustment for apolipoprotein B. Additional Model 4 was adjusted as in the main model, with additional adjustment for LDL cholesterol, HDL cholesterol and total triglyceride. Additional Model 5 was adjusted as in the main model, with additional adjustment for LDL cholesterol, HDL cholesterol and total triglyceride minus HDL-TG. CAD, coronary artery disease; CI, confidence intervals; CVD, cardiovascular disease; HDL, high density lipoprotein; HDL-TG, Triglycerides in HDL; HR, hazard ratio; LDL, low-density lipoprotein.

Figure 4A presents the association between HDL-TG and incident CAD events. After adjusting for a comprehensive set of confounders, the observational analysis revealed a 6% increase in CAD risk for each 1 SD increment in HDL-TG levels (HR: 1.066, 95% CI: 1.052–1.080, *P* < 0.001). Compared to individuals in the lowest tertile (1^st^ - 50^th^ percentile) of HDL-TG, those in the middle tertile (51^st^ - 95^th^ percentile) exhibited a significantly higher risk of CAD events (HR: 1.109, 95% CI: 1.078–1.140, *P* < 0.001). This risk further increased for individuals in the highest tertile (96^th^ - 100^th^ percentile) of HDL-TG levels (HR: 1.197, 95% CI: 1.132–1.266, *P* < 0.001) (Fig. 4A, Supplemental Table 9). Figure 4B displays the results of an RCS analysis examining the association between HDL-TG levels and CAD event rates. The RCS curve demonstrated a linear relationship, suggesting a continuous increase in CAD risk with increasing HDL-TG levels (*P* for nonlinear = 0.279, after fully adjusted confounders).

The results of additional analysis adjusting for various biomarkers showed that the association remained statistically significant (all *P* < 0.05) even after further adjustment for CRP; HDL-P and average diameter; ApoB; LDL-C, HDL-C, and total TG or total TG minus HDL-TG levels, individually (Fig. 4C).

To examine whether the association between HDL-TG and CAD events persists across different baseline cardiovascular risk profiles, we stratified our analysis by predetermined CVD risk categories (calculated using the FRS risk score). Figure 4D shows that higher HDL-TG levels were consistently associated with an increased risk of CAD events, even among individuals traditionally considered to be at low overall cardiovascular risk. Specifically, in the low CVD risk group, participants in the middle (51^st^ - 95^th^ percentile) and highest tertile (96^th^ - 100^th^ percentile) of HDL-TG levels exhibited a 19% (HR: 1.191, 95% CI: 1.127–1.259, *P* < 0.001) and 29% (HR: 1.287, 95% CI: 1.155–1.435, *P* < 0.001) higher risk of CAD events, respectively, compared to the reference group (1^st^ - 50^th^ percentile). Similarly, in the high CVD risk group, the middle and highest tertile of HDL-TG levels showed a 5% (HR: 1.054, 95% CI: 1.021–1.089, *P* = 0.001) and 18% (HR: 1.183, 95% CI: 1.104–1.267, *P* < 0.001) increase in CAD risk, respectively.

### Discordance between HDL-TG and ApoB, LDL-C, and total-TG in CAD risk

Given prior research indicating ApoB as a primary determinant of CAD risk (25), and the well-established importance of LDL-C and TG, we conducted a discordance analysis to further elucidate the independent and potentially additive contributions of elevated HDL-TG, ApoB, LDL-C, and total-TG to CAD risk.

**Fig. 5:**
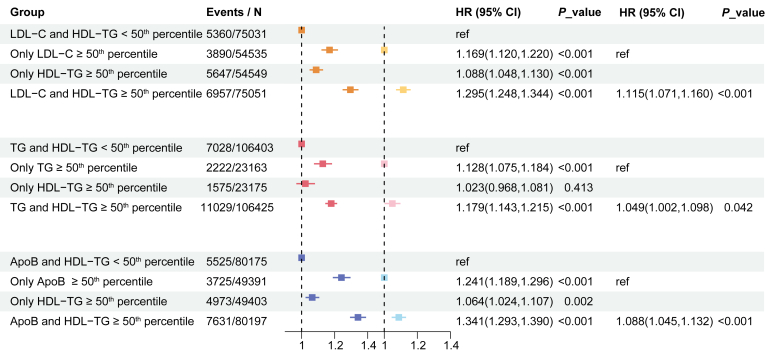
Discordance Between HDL-TG and ApoB, LDL-C, and total-TG on risk of CAD. Model adjusted for age, sex, and ethnic background, overall health rating, education qualifications, smoking status, alcohol drinker status, assessment centre, Townsend deprivation index, history of diabetes, history of hypertension, lipid-lowering therapy, antihypertensive therapy, insulin therapy, systolic blood pressure, glucose, and body mass index. ApoB, ApolipoproteinB; CAD, coronary artery disease; CI, confidence intervals; HDL-TG, Triglycerides in high-density lipoprotein; HR, hazard ratio; LDL-C, low-density lipoprotein cholesterol; TG, Triglycerides.

Figure 5 compares the impact of HDL-TG and each of the biomarkers (ApoB, LDL-C, and total-TG) on CAD risk by stratifying participants into four groups based on the 50^th^ percentiles of each biomarker. Individuals with higher HDL-TG levels consistently exhibited a higher risk of CAD compared to those with both HDL-TG and the corresponding biomarker levels below the 50^th^ percentile, or compared to those with only the corresponding biomarker above the 50^th^ percentile, regardless of the specific status of ApoB or LDL-C (all *P* < 0.05, after fully adjusted confounders). In contrast, the discordance analysis between TG and HDL-TG showed that participants with elevated HDL-TG alone did not have a significantly increased risk of CAD compared to those with both TG and HDL-TG levels below the 50^th^ percentile (HR: 1.023, 95% CI: 0.968–1.081, *P* = 0.413, after fully adjusted confounders). However, individuals with both elevated HDL-TG and TG levels exhibited a significantly higher risk of incident CAD, regardless of the total TG levels (all *P* < 0.05, after fully adjusted confounders). In addition, Supplemental Fig. 2 further presents the CAD risk of individuals with extremely high levels of these biomarkers, as stratified by the 80^th^ percentile.

### Causal inference of HDL-TG with prevalence of CAD: MR evidence

**Fig. 6:**
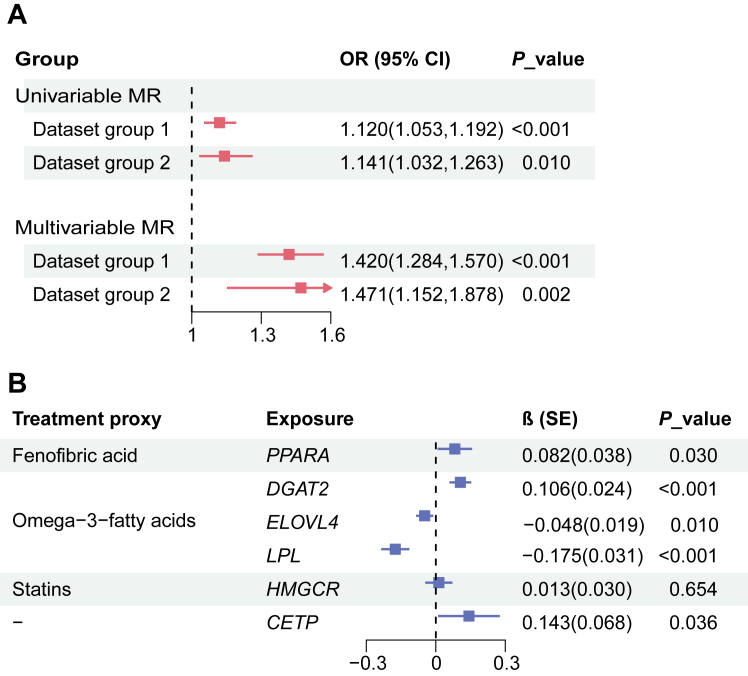
Mendelian randomization results. A: Association of HDL-TG with CAD Analyzed by Univariable and Multivariable MR. B: Associations of Known Targets of Lipid-lowering Genetic Variants in Drug Target Gene Loci with the Risk of HDL-TG Elevation. MR study: Causal estimates are obtained using the inverse-variance weighted method. Multivariate adjusted for HDL_C, LDL_C and Total_TG. *CETP*, Cholesteryl Ester Transfer Protein; *DGAT2*, Diacylglycerol O-acyltransferase 2; *ELOVL4*, Elongation of very long chain fatty acids protein 4; HDL-TG, Triglycerides in HDL; *HMGCR*, 3-Hydroxy-3-Methylglutaryl-CoA Reductase; *LPL*, Lipoprotein lipase; *PPARA*, Peroxisome proliferator-activated receptor alpha; SE, standard errors.

Figure 6A and Supplemental Table 10 present the results of two-sample MR analysis, which investigated the causal association between HDL-TG and CAD risk. Both univariable and multivariable MR analysis, employing various MR methods and two independent datasets, consistently demonstrated a significant positive association between genetically predicted higher HDL-TG levels and increased CAD risk. In the univariable MR analysis, the IVW method yielded ORs of 1.120 (95% CI: 1.053–1.192, *P* < 0.001) and 1.141 (95% CI: 1.032–1.263, *P* = 0.010) for dataset groups 1 and 2, respectively (Fig. 6A). The weighted median and weighted mode methods yielded similar effect estimates, further supporting the robustness of the causal association (Supplemental Table 10). Multivariable MR analysis, further adjusting for HDL-C, LDL-C, and total TG, confirmed the independent causal effect of HDL-TG on CAD risk with ORs of 1.420 (95% CI: 1.284–1.570, *P* < 0.001) and 1.471 (95% CI: 1.152–1.878, *P* = 0.002) for dataset groups 1 and 2, respectively (Fig. 6A).

### Associations of lipid-lowering medications with HDL-TG levels through drug-target MR analysis

To explore the potential impact of existing lipid-lowering medications on HDL-TG levels, we conducted drug-target MR studies, with results presented in Fig. 6B and Supplemental Table 11. This included medications targeting hypertriglyceridemia, lowering LDL-C levels, and raising HDL-C levels. For OM3-FAs, using *DGAT2* and *LPL* as a proxy, MR analysis suggested a potential association with HDL-TG levels (IVW β (SE) for *DGAT2*: 0.106 (0.024), *P* < 0.001; *LPL*: −0.175 (0.031), *P* < 0.001), confirmed by both weighted median and mode methods. However, *ELOVL4* and *PPARA* showed a potential association using IVW but were not replicated by other MR methods. Statin treatment (via *HMGCR*) did not reveal a significant effect on HDL-TG levels. Interestingly, our results suggest a potential effect of *CETP* on HDL-TG levels (IVW β (SE): 0.143 (0.068), *P* = 0.036), which, despite the current absence of approved *CETP*-targeting drugs, may indicate a future therapeutic direction worth further exploration.

### Additional analyses

Supplementary analyses demonstrated that VLDL and LDL subcomponents were positively associated with CAD risk, with LDL-TG showing a significant causal association in MR analysis. However, TG in VLDL and LDL did not exhibit the same level of specificity as HDL-TG in relation to CAD. These results are detailed in Supplemental Tables 12–18 and Supplemental Figs. 3 and 4.

The HDL-TG/HDL-C ratio showed a significant positive association with CAD risk (HR: 1.108, 95% CI: 1.095–1.121, *P* < 0.001, after fully adjusted confounders) (Supplemental Table 19). The Akaike Information Criterion (AIC) and Bayesian Information Criterion (BIC) values were lower for the HDL-TG/HDL-C ratio model compared to the HDL-TG-only model, suggesting a better balance between model fit and complexity, while the C-index also showed slightly improved discrimination ability (Supplemental Table 20).

Additionally, MR analysis was conducted to evaluate the causal relationship between HDL-C and CAD using two independent datasets. In Dataset 1, HDL-C demonstrated a significant inverse causal association with CAD (OR: 0.883, 95% CI: 0.839–0.928, *P* < 0.001 in IVW analysis). However, in Dataset 2, the association was not significant (OR: 0.994, 95% CI: 0.815–1.214, *P* = 0.956 in IVW analysis) (Supplemental Table 21). The inconsistency across datasets suggests that the evidence for a causal relationship between HDL-C and CAD is inconclusive within our study.

## Discussion

This comprehensive study, integrating data from a large prospective cohort and MR analysis, demonstrated that elevated serum TG was associated with adverse alterations in HDL size and composition, characterized by reduced particle diameter, decreased concentrations of major HDL components (cholesterol, phospholipids, and cholesteryl esters), and increased HDL-TG, and these changes may be associated with increased CAD risk. Furthermore, we identified an independent and positive association between HDL-TG levels and incident CAD events, even after adjusting for established cardiovascular risk factors and other lipid parameters. Elevated HDL-TG levels remained associated with an increased risk of CAD events even in populations traditionally considered to have a lower overall cardiovascular risk. MR analysis provided evidence supporting a causal role of HDL-TG in CAD development. Finally, drug-target MR analysis suggested a potential association between OM3-FA supplementation and lower HDL-TG levels. This analysis may offer potential insights into reducing CAD risk associated with alterations in HDL size and composition.

Our findings align with previous studies reporting associations between elevated TG and changes in HDL composition. For example, Ibi *et al.*, in a GWAS of postprandial TG response, identified the rs7350789-A variant in *LIPC*, encoding hepatic lipase (HL), to be associated with increased TG content in nearly all HDL subfractions and decreased HDL diameter (19). Amigó *et al*. and Kontush *et al.* demonstrated that elevated serum TG levels, observed in both hypertriglyceridemic and postprandial states, are associated with altered HDL particle characteristics—including an increased proportion of hydrophobic core lipids (primarily esterified cholesterol and TG) on the HDL surface, TG enrichment coupled with cholesteryl ester depletion in the HDL core, and reduced particle size (48, 49). However, our study extends existing knowledge by specifically focusing on the crucial role of HDL-TG in the association of HDL with CAD. Previous research has primarily focused on measuring HDL function through cholesterol efflux capacity or CETP activity and advocated for improving CVD risk by raising HDL-C levels or modulating CETP activity, yet these trials have proven unsuccessful (5, 6, 8, 10, 11, 12, 13). Our study emphasizes the importance of considering HDL composition, particularly TG content, to understand its impact on CVD risk. By leveraging a large prospective cohort and MR analyses, coupled with robust confounder adjustment and assessment of linear relationships, we were able to assess the independent and causal association between HDL-TG and CAD risk, providing stronger evidence than previous observational studies (22, 23, 24, 25, 26). Additionally, for the first time, we explored the potential impact of existing lipid-lowering medications on HDL-TG levels, identifying OM3-FA supplementation as a potential therapeutic target based on drug-target MR analysis. This comprehensive study offers novel insights into the assessment and treatment of TG-induced alterations in HDL size and composition, highlighting the potential of HDL-TG as a crucial factor in ASCVD development and a promising target for therapeutic interventions.

The mechanisms by which elevated TG and HDL-TG promote CAD development are complex and multifaceted. One potential pathway involves specific structural alterations in HDL particles when they become enriched in TG. As reported by Amigó *et al.*, this includes a partial extrusion of hydrophobic core lipids toward the surface of the HDL particle, a phenomenon described as “herniation” (49). This structural remodeling can impair normal HDL functionality by disrupting the molecular interactions between lipoproteins, enzymes, and cell membranes, and also by affecting the conformation of apolipoproteins (48, 50). The reduction in HDL particle size observed in our study exacerbates this “herniation” effect as smaller particles are subject to stricter spatial constraints, forcing core hydrophobic lipids to migrate to the surface (49). Beyond these direct structural effects, alterations in HDL lipid composition may also significantly affect functionality through two primary pathways: First, CETP-mediated core lipid exchange: CETP facilitates the exchange of TG from TG-rich lipoproteins to HDL particles, particularly in the context of hypertriglyceridemia or postprandial TG elevation (9, 48). Second, LPL-mediated transfer of surface remnants: LPL hydrolyzes TG-rich lipoproteins, such as VLDL and chylomicrons, generating surface remnants that are taken up by HDL (51). Under hypertriglyceridemic conditions, the LPL-mediated pathway is further amplified, leading to the formation of TG-enriched HDL particles (51). These compositional changes, combined with the aforementioned structural effects, not only impair cholesterol efflux and antioxidant activity but also render these particles more susceptible to modification by HL, further diminishing their cardioprotective capacity (9, 19, 48, 52, 53).

Additionally, previous studies on HDL proteomics have demonstrated that postprandial TG accumulation is associated with alterations in the HDL protein composition (54). For example, such changes include the release of PCSK9 and apoC3 from HDL, as well as an increase in inflammation-related proteins (54). When PCSK9 is released from HDL, it may bind more effectively to LDL receptors and promote their degradation, thereby increasing circulating atherogenic lipoproteins (54). Similarly, apoC3, known to inhibit LPL activity—which is responsible for the hydrolysis of plasma TG—can accumulate in the circulation once released from HDL, further suppressing LPL activity and resulting in elevated TG levels (54). The concomitant increase in apoC3 and other inflammation-related proteins may exacerbate inflammatory responses, creating an environment that promotes the formation and progression of atherosclerotic plaques.

Our findings support the concept that OM3-FA, a commonly prescribed medication for the treatment of severe hypertriglyceridemia (55), may potentially influence HDL-TG levels, as indicated by drug-target MR analysis. Previous research has demonstrated that saturated fatty acids can reduce the anti-inflammatory potential of HDL, impair endothelial function, and potentially promote atherogenesis (56). Conversely, polyunsaturated fatty acids, such as those found in OM3-FA, may enhance HDL's anti-inflammatory activity, contributing to cardiovascular protection (56). Calabresi *et al.* found that Omacor, a concentrated formulation of omega-3 polyunsaturated fatty acids, not only decreased plasma TG levels but also increased HDL2 cholesterol and paraoxonase activity, an antioxidant enzyme associated with HDL (57). Previous studies on icosapent ethyl—a specific OM3-FA formulation—have also underscored this potential. For example, the REDUCE-IT trial demonstrated that, in patients with hypertriglyceridemia, treatment with icosapent ethyl significantly reduced the risk of ASCVD events and favorably modulated various biomarkers, including HDL levels (58). These findings may partly explain the observed potential association between OM3-FA supplementation and HDL-TG levels. However, due to the high correlation between total TG and HDL-TG, further research is needed to elucidate the precise mechanisms underlying OM3-FA's impact on HDL-TG, including any differences compared to its effect on lowering TG, and explore the efficacy and safety of OM3-FA therapy in reducing CAD risk in individuals with elevated HDL-TG levels.

Our study has several limitations. First, despite comprehensive adjustments in our observational analyses for a wide range of potential confounders and the application of various methods to detect and adjust for horizontal pleiotropy in our MR analyses—including adjustments for total TG (or total TG minus HDL-TG), LDL-C, and HDL-C—the possibility that residual confounding, unaccounted pleiotropic effects, or mediation by overall TG levels or TG in other lipoproteins may partially influence the observed association between HDL-TG and CAD risk cannot be completely ruled out. Second, our study utilized metabolomics profiling via the Nightingale platform. While this platform has been widely applied in large-scale epidemiological studies, concerns have been raised regarding potential underestimation of LDL and HDL particle concentrations and inconsistencies in metabolic relationships, which may introduce measurement bias (59). However, because our analyses focused on the relative associations between HDL-TG and CAD risk—rather than absolute particle concentrations—these biases may have been mitigated. Moreover, the robust positive association we observed between HDL-TG and CAD risk, which was distinct from other HDL subcomponents, strengthens confidence in our findings. Nevertheless, given the proprietary nature of the Nightingale algorithms and the potential for systematic measurement errors (59), our results should be interpreted with caution. Future studies using alternative metabolomics platforms and independent validation cohorts are warranted to confirm our findings. Third, our drug-target MR analysis focused on medications associated with the treatment of hypertriglyceridemia, the lowering of LDL-C levels, and the raising of HDL-C levels, based on a limited number of pharmacological targets selected according to the availability of cis-eQTL data. Fourth, while our additional analyses indicate that the HDL-TG/HDL-C ratio may serve as a promising cardiovascular risk marker, the lack of genome-wide association study (GWAS) summary data for this ratio precluded MR validation of its causal relationship with CAD. Future research is needed to evaluate the potential role and biological significance of this promising ratio.

In conclusion, our comprehensive study reveals that elevated serum TG is associated with detrimental alterations in HDL size and composition, potentially contributing to an increased risk of CAD. We identified HDL-TG as an independent and potentially causal risk factor for CAD. Drug-target MR analysis suggests that OM3-FA supplementation may be associated with a potential reduction in HDL-TG levels. Further research is warranted to elucidate the precise mechanisms underlying these observations and to explore therapeutic strategies targeting HDL-TG for ASCVD prevention and treatment.

## Data availability

Researchers interested in accessing the datasets utilized in this observational study can initiate the process by visiting the UK Biobank website. By submitting an application inclusive of a research protocol summary and requested data fields, they can apply for access. Upon approval by the UK Biobank management team and payment of relevant fees, researchers will gain entry to the database. Additionally, the datasets employed in this MR study are publicly available in summary form. They can be located in referenced papers, the IEU OpenGWAS Project repository, or the GWAS Catalogue repository. Data availability is subject only to constraints imposed by the corresponding data committee.

## Supplemental data

This article contains supplemental data.

## Conflict of interests

The authors declare that they have no conflicts of interest with the contents of this article.
