# Supplementary material for: Elevated high-density lipoprotein triglycerides increase atherosclerotic risk
Source: J Lipid Res. 2025 Mar 29;66(5):100791. doi: 10.1016/j.jlr.2025.100791 (PMC12088758; doi:10.1016/j.jlr.2025.100791)
Supplement: Supplementary Materials [file mmc1.docx]

**Supplementary Materials**

**Elevated High-Density Lipoprotein Triglycerides Increase Atherosclerotic Risk**

*Liu et al.*

**Supplementary Methods**

**Supplementary Tables**

**Supplemental Table 1.** Two-sample MR Data Sources.

**Supplemental Table 2.** CAD definition used in this study.

**Supplemental Table 3.** Assessment of Covariates in the UK Biobank.

**Supplemental Table 4.** Detailed information on SNPs included in MR analysis (related to Supplemental Table 10).

**Supplemental Table 5.** Instruments for Drug Target Genes (related to Supplemental Table 11).

**Supplemental Table 6.** Correlation analysis between triglycerides and HDL Subcomponents.

**Supplemental Table 7.** Association between HDL-P and Incident Coronary Artery Disease Events across Triglyceride Levels.

**Supplemental Table 8.** Association between HDL-C and Incident Coronary Artery Disease Events across Triglyceride Levels.

**Supplemental Table 9.** Association of Triglycerides in HDL with Incident CAD Events by Percentile Ranges.

**Supplemental Table 10.** Causal effects of Triglycerides in HDL on CAD using different methods.

**Supplement Table 11.** Mendelian Randomization Associations of Known Targets of Lipid-lowering Genetic Variants in Drug Target Gene Loci with the Risk of HDL-TG Elevation.

**Supplemental Table 12.** Baseline Characteristics of VLDL Subcomponents by Grouping of Triglyceride Levels.

**Supplemental Table 13.** Baseline Characteristics of LDL Subcomponents by Grouping of Triglyceride Levels.

**Supplemental Table 14.** Association of Subcomponents in VLDL with Incident Coronary Artery Disease Events.

**Supplemental Table 15.** Association of Subcomponents in LDL with Incident Coronary Artery Disease Events.

**Supplemental Table 16.** Causal effects of Triglycerides in VLDL on CAD using different methods.

**Supplemental Table 17.** Causal effects of Triglycerides in LDL on CAD using different methods.

**Supplemental Table 18.** Causal effects of Triglycerides in LDL on CAD using multivariate mendelian randomization analysis method.

**Supplemental Table 19.** Association of HDL-TG/HDL-C ratio with Incident Coronary Artery Disease Events.

**Supplemental Table 20.** Comparison of Model Performance Metrics Between HDL-TG and HDL-TG/HDL-C Models for Predicting CAD Risk.

**Supplemental Table 21.** Causal effects of HDL-C on CAD using different methods.

**Supplementary Figure**

**Supplemental Figure 1.** Flow chart of participants.

**Supplemental Figure 2.** Discordance Between HDL-TG and ApoB, LDL-C, and total-TG on Risk of CAD, as stratified by the 80^th^ percentile.

**Supplemental Figure 3.** Association of Subcomponents in VLDL/LDL with Incident Coronary Artery Disease Events across Different Levels of VLDL/LDL Particle Diameter.

**Supplemental Figure 4.** Association between LDL-P/ LDL-C and Incident Coronary Artery Disease Events across Triglyceride Levels.

**Supplementary Methods**

**Section 1: Study design and data sources**

**Study Design**

Initially, to assess the impact of elevated serum triglycerides (TG) on high-density lipoprotein (HDL) size and composition and their potential association with coronary artery disease (CAD) risk, we conducted descriptive analysis, stratifying participants based on serum TG levels to evaluate changes in HDL particle diameter and subcomponent characteristics. Then, we evaluated the relationship between HDL particle concentration (HDL-P), HDL cholesterol (HDL-C), and incident CAD events across different TG levels. Secondly, we investigated the association between HDL subcomponents and incident CAD events across various levels of HDL particle diameter. Thirdly, we examined the independent association between TG in HDL (HDL-TG) and incident CAD through both observational analysis and two-sample Mendelian Randomisation (MR) studies. Additionally, we assessed the robustness of this association after adjusting for additional confounding factors, such as C-reactive protein (CRP); HDL particle number and average diameter; apolipoprotein B (ApoB); low-density lipoprotein cholesterol (LDL-C), HDL-C, and total TG levels; and LDL-C, HDL-C, and total TG minus HDL-TG, respectively. Moreover, we further explored the discordance between HDL-TG and ApoB, HDL-TG and LDL-C, and HDL-TG and TG concerning the risk of CAD, as well as the association of HDL-TG with incident CAD events stratified by general cardiovascular diseases (CVD) risk scores. Further, we employed restricted cubic spline (RCS) analysis to investigate whether this association exhibits a linear relationship. Finally, we investigated the potential associations of known targets of lipid-lowering genetic variants in drug target gene loci with the risk of HDL-TG elevation.

**Data Sources**

Observational Analysis: UK Biobank database contains extensive phenotypic and genotypic information about its participants, including data from questionnaires, biological measurements, lifestyle indicators, biomarkers in blood and urine, and longitudinal follow-up data for a wide range of health-related outcomes.^1^

Two-sample MR Analysis: Briefly, we employed two sets of datasets to explore and validate the potential causal association between HDL-TG and the prevalence of CAD. For dataset group 1, we utilized genome-wide association studies (GWAS) of metabolic biomarkers measured by high-throughput nuclear magnetic resonance (NMR) spectroscopy in the UK Biobank, primarily in individuals of European ancestry, as the exposure data source (https://gwas.mrcieu.ac.uk/datasets/?gwas_id__icontains=met-d). NMR measurements in the UK Biobank were inverse rank-order normalized. The outcome data were derived from a meta-analysis of GWAS conducted on 185,000 CAD cases and controls of European ancestry.^2^ In dataset group 2, we expanded our exposure and outcome populations to include mixed-ancestry populations. The exposure data source consisted of GWAS for serum metabolomics, utilizing Nightingale NMR to quantify 230 serum metabolic biomarkers from 37,359 INTERVAL participants.^3^ The outcome data source was derived from a GWAS analysis of CAD conducted by the CARDIoGRAMplusC4D Consortium, which included 63,746 CAD cases and 130,681 controls.^4^

**Section 2: Observational Analysis**

**Classification of total TG**

The five groups are as follows: (1) low-normal TGs: <100 mg/dL (1.129 mmol/L); (2) high-normal TGs: 100-149 mg/dL (1.129-1.684 mmol/L); (3) borderline hypertriglyceridemia: 150-199 mg/dL (1.684-2.257 mmol/L); (4) moderate hypertriglyceridemia: 200-499 mg/dL (2.257-5.644 mmol/L); (5) severe hypertriglyceridemia: ≥500 mg/dL (5.644 mmol/L).

**NMR-based metabolic biomarker profiling platform**

This platform allows for the measurement of 251 metabolic biomarkers in EDTA plasma samples obtained from approximately 280,000 UK Biobank participants. Details regarding the NMR platform and experimental procedures can be found in the documentation of the UK Biobank study (https://biobank.ndph.ox.ac.uk/ukb/label.cgi?id=220).

**Ascertainment of CAD**

CAD diagnoses were derived from various sources including hospital inpatient data, mortality records, primary care records, and participant-reported medical conditions at baseline assessment. These records were meticulously mapped to ICD10 codes by the UK Biobank team. Detailed information regarding the specific mapping methodology is available through the UK Biobank platform (Resource Number 593).

**Calculation of the general cardiovascular risk score**

Sex-specific multivariable risk functions ("general CVD" algorithms) were derived that incorporated age, total cholesterol, HDL-C, systolic blood pressure (SBP), treatment for hypertension, smoking, and diabetes status.^5^ The FRS, developed by the Framingham Heart Study in the United States, is widely used globally, including in Canada, to assess general CVD risk and the risk of individual CVD events (CAD, cerebrovascular disease, peripheral artery disease, and heart failure).^5,6^

**Section 3: Two-Sample MR Analysis**

**Genetic instruments**

MR, based on three assumptions, is less susceptible to measurement errors, confounding factors, and reverse causal relationships compared to traditional regression methods: (1) genetic variants have a strong correlation with exposure (relevance); (2) genetic variants are independent of confounding factors in the exposure-outcome relationship (independence); and (3) genetic variants do not affect the outcome through pathways other than the exposure (exclusion restriction). In this study, we selected single-nucleotide polymorphisms (SNPs) strongly associated with exposure as instrumental variables based on the following criteria: (1) SNPs associated with exposure at the genome-wide statistical significance level (*P* < 5×10^-8^); (2) clustering SNPs within a 10,000 kB window to an linkage disequilibrium (LD) threshold of *R*^2^ < 0.1 using 1000 Genomes European Ancestry as a reference panel. For SNPs in LD, we chose the variant with the lowest *P*-value; and (3) an *F* statistic ≥ 10 to reduce the risk of weak instrument bias.

The *F* statistic and *R^2^* were computed to assess the strength of instrumental variables and their explanatory power for the phenotype. The *R^2^* was calculated using the approximation formula *R^2^* = 2EAF (1-EAF) β^2^, where EAF represents the effect allele frequency and β denotes the estimated genetic effect on exposure.^7^ The formula for the *F* statistic is as follows: *F* = *R^2^*(N-k-1)/k(1-*R^2^*), where N is the sample size and k is the number of instrumental variables.^8^ An *F* statistic ≥10 indicates a relatively low risk of weak instrument bias.^9^ All SNPs used as instrumental variables are listed in **Supplemental Table 4**.

**Univariable MR**

We first conducted univariable MR analysis to explore the association between genetically predicted HDL-TG and CAD. We employed several different methods to ensure the robustness of the results: 1. the inverse variance weighted (IVW) method, which is a robust MR approach assuming valid instrumental variables and balanced pleiotropy.^10^ 2. weighted median method, assessing for invalid instrument bias, assuming that more than 50% of the weight is provided by valid SNPs.^11^ 3. MR-Egger regression, allowing instruments to exhibit horizontal pleiotropy, under the premise that each genetic variant's association with exposure is independent of its pleiotropic effects.^12^ 4. weighted mode method, having robustness in handling horizontal pleiotropy by weighting the modes based on SNP weights. 5. MR-Egger intercept, estimating horizontal pleiotropy, along with Cochrane’s Q statistic to test for heterogeneity; if *P* ≤ 0.5, the multivariable random-effects IVW method is applied for analysis.^10,12^ 6. MR pleiotropy residual sum and outlier (MR-PRESSO) method, identifying and removing outlier SNPs that may exhibit horizontal pleiotropy to correct the estimated causal effect.^13^

**Multivariable MR**

We further employed a multivariable MR approach, permitting the simultaneous assessment of multiple risk factors,^14^ to evaluate the potential causal association between genetically predicted HDL-TG and CAD after adjusting for HDL-C, LDL-C, and total TG.

**Section 4: Additional Analysis**

We conducted supplementary analyses to further explore the associations between very low-density lipoprotein (VLDL) and LDL subcomponents and CAD. Subcomponent concentrations and particle diameters of VLDL and LDL were analyzed across TG stratification groups. Cox proportional hazards models were employed to evaluate the associations between these subcomponents and CAD, with covariate adjustments consistent with the main analysis. MR analysis was also performed to explore the potential causal effects of LDL-TG and VLDL-TG on CAD.

In addition, we expanded our investigation to explore the association between the HDL-TG/HDL-C ratio and incident CAD. Cox proportional hazards models were utilized to assess the risk, with adjustment variables consistent with the main analysis. Model performance metrics, including Akaike Information Criterion (AIC), Bayesian Information Criterion (BIC), and C-index, were employed to compare the predictive ability of the models featuring HDL-TG and HDL-TG/HDL-C. However, due to the unavailability of GWAS summary data for the HDL-TG/HDL-C ratio, MR analysis could not be conducted to explore its causal relationship with CAD.

Finally, to assess the causal relationship between HDL-C and CAD, we performed MR analysis using two separate datasets, following the approach used for HDL-TG and CAD in the main analysis, providing additional insight into the role of HDL-C in CAD risk.

**References**

1. Walsh JW, Hoffstad OJ, Sullivan MO, Margolis DJ. Association of diabetic foot ulcer and death in a population-based cohort from the United Kingdom. Diabet Med. 2016 Nov;33(11):1493–8.

2. Nikpay M, Goel A, Won HH, Hall LM, Willenborg C, Kanoni S, et al. A comprehensive 1,000 Genomes-based genome-wide association meta-analysis of coronary artery disease. Nat Genet. 2015 Oct;47(10):1121–30.

3. Xu Y, Ritchie SC, Liang Y, Timmers PRHJ, Pietzner M, Lannelongue L, et al. An atlas of genetic scores to predict multi-omic traits. Nature. 2023 Apr;616(7955):123–31.

4. Deloukas P, Kanoni S, Willenborg C, Farrall M, Assimes TL, Thompson JR, et al. Large-scale association analysis identifies new risk loci for coronary artery disease. Nat Genet. 2013 Jan;45(1):25–33.

5. D’Agostino RBS, Vasan RS, Pencina MJ, Wolf PA, Cobain M, Massaro JM, et al. General cardiovascular risk profile for use in primary care: the Framingham Heart Study. Circulation. 2008 Feb;117(6):743–53.

6. Pearson GJ, Thanassoulis G, Anderson TJ, Barry AR, Couture P, Dayan N, et al. 2021 Canadian Cardiovascular Society Guidelines for the Management of Dyslipidemia for the Prevention of Cardiovascular Disease in Adults. Can J Cardiol. 2021 Aug;37(8):1129–50.

7. Papadimitriou N, Dimou N, Tsilidis KK, Banbury B, Martin RM, Lewis SJ, et al. Physical activity and risks of breast and colorectal cancer: a Mendelian randomisation analysis. Nat Commun. 2020 Jan;11(1):597.

8. Burgess S, Thompson SG. Avoiding bias from weak instruments in Mendelian randomization studies. Int J Epidemiol. 2011 Jun;40(3):755–64.

9. Palmer TM, Lawlor DA, Harbord RM, Sheehan NA, Tobias JH, Timpson NJ, et al. Using multiple genetic variants as instrumental variables for modifiable risk factors. Stat Methods Med Res. 2012 Jun;21(3):223–42.

10. Bowden J, Del Greco M F, Minelli C, Davey Smith G, Sheehan N, Thompson J. A framework for the investigation of pleiotropy in two-sample summary data Mendelian randomization. Stat Med. 2017 May;36(11):1783–802.

11. Bowden J, Davey Smith G, Haycock PC, Burgess S. Consistent Estimation in Mendelian Randomization with Some Invalid Instruments Using a Weighted Median Estimator. Genet Epidemiol. 2016 May;40(4):304–14.

12. Burgess S, Thompson SG. Interpreting findings from Mendelian randomization using the MR-Egger method. Eur J Epidemiol. 2017 May;32(5):377–89.

13. Verbanck M, Chen CY, Neale B, Do R. Detection of widespread horizontal pleiotropy in causal relationships inferred from Mendelian randomization between complex traits and diseases. Nat Genet. 2018 May;50(5):693–8.

14. Sanderson E, Davey Smith G, Windmeijer F, Bowden J. An examination of multivariable Mendelian randomization in the single-sample and two-sample summary data settings. Int J Epidemiol. 2019 Jun;48(3):713–27.

**Supplementary Tables**

**Supplemental Table 1. Two-sample MR Data Sources.**

1. **GWAS summary data.**

| **Phenotype** | | **Major population** | **Sample size** | **Consortium** | **GWAS data sourece** |
| --- | --- | --- | --- | --- | --- |
| Dataset group 1 | Metabolic biomarkers | European-ancestry | 115,078 | UK Biobank | Nightingale Health Plc; biomarker quantification version 2020* |
|  | Coronary artery disease | European-ancestry | 141,217 | Meta-analysis | DOI: 10.1038/ng.3396 |
| Dataset group 2 | Metabolic biomarkers | Mixed | 37,359 | INTERVAL study | DOI: 10.1038/s41586-023-05844-9 |
|  | Coronary artery disease | Mixed | 194,427 | CARDIoGRAMplusC4D | DOI: 10.1038/ng.2480 |

* Data are available on genetic associations with 249 circulating metabolites measured by high-throughput NMR spectroscopy from https://gwas.mrcieu.ac.uk/datasets/?gwas_id__icontains=met -d.

1. **eQTL summary data.**

| **Data set** | **Tissue** | **Major population** | **Sample size** | **Number of SNPs** | **No. of probes and/or genes** |
| --- | --- | --- | --- | --- | --- |
| eQTLGen | Blood and peripheral blood mononuclear cell | European-ancestry | 31,684 | 10,317 | 19,942 |

The eQTLGen summary data are available through application to the eQTLGen consortium.

1. **Gene information for drug target MR.**

| **Treatment proxy** | **Target** | **Ensembl** | **Chromosome** | **Genomic Locations (GRCh37/hg19)** |
| --- | --- | --- | --- | --- |
| Fenofibric acid | *PPARA* | ENSG00000186951 | 22 | 46,546,429-46,639,652 |
| Omega-3-carboxylic acids | *DGAT2* | ENSG00000062282 | 11 | 75,479,823-75,512,579 |
|  | *LPL* | ENSG00000175445 | 8 | 19,796,764-19,824,770 |
|  | *ELOVL4* | ENSG00000118402 | 6 | 80,624,531-80,657,270 |
| Statins | *HMGCR* | ENSG00000113161 | 5 | 74,632,154-74,657,929 |
| - | *CETP* | ENSG00000087237 | 16 | 56,995,762-57,017,757 |

Abbreviation: MR, Mendelian randomisation; GWAS, genome-wide association study; *PPARA*, Peroxisome proliferator-activated receptor alpha; *DGAT2*, Diacylglycerol O-acyltransferase 2; *ELOVL4*, Elongation of very long chain fatty acids protein 4; *LPL*, Lipoprotein lipase; *HMGCR*, 3-Hydroxy-3-Methylglutaryl-CoA Reductase; *CETP*, Cholesteryl Ester Transfer Protein; SNPs, single-nucleotide polymorphisms.

**Supplemental Table 2. CAD definition used in this study.**

| **Diagnosis** | **UK Biobank field ID** | **ICD10 Code** |
| --- | --- | --- |
| Angina pectoris | 131296 | I20 |
| Myocardial infarction | 131298 | I21 |
| Subsequent myocardial infarction | 131300 | I22 |
| Certain current complications following acute myocardial infarction | 131302 | I23 |
| Other acute ischaemic heart diseases | 131304 | I24 |
| Chronic ischaemic heart disease | 131306 | I25 |

Abbreviation: CAD, coronary artery disease; ICD, International Classification of Diseases.

**Supplementary Table 3. Assessment of Covariates in the UK Biobank.**

| **Covariates** | **UK Biobank field ID** |
| --- | --- |
| Sex | 31 |
| UK Biobank assessment centre | 54 |
| Overall health rating | 2178 |
| Diabetes diagnosed by doctor | 2443 |
| Started insulin within one year diagnosis of diabetes | 2986 |
| Diastolic blood pressure | 4079 |
| Systolic blood pressure | 4080 |
| Education qualifications | 6138 |
| Vascular/heart problems diagnosed by doctor | 6150 |
| Medication for cholesterol, blood pressure, diabetes | 6153/6177 |
| Smoking status | 20116 |
| Alcohol drinker status | 20117 |
| Ethnic background | 21000 |
| Body mass index | 21001 |
| Age at recruitment | 21022 |
| Townsend deprivation index at recruitment | 22189 |
| C-reactive protein | 30710 |
| Glucose | 30740 |

**Supplemental Table 4. Detailed information on SNPs included in MR analysis (related to Supplemental Table 10).**

| **Dataset Group** | **SNP** | **effect allele** | **other allele** | **Beta.exposure** | **Beta.outcome** | **Eaf.exposure** | **Eaf.outcome** | **chr** | **position** | **Samplesize.outcome** | **Pval.outcome** | **Se.outcome** | **Pval.exposure** | **Samplesize.exposure** | **Se.exposure** | **MAF** | ***R^2^*** | ***F*-stastic** |
| --- | --- | --- | --- | --- | --- | --- | --- | --- | --- | --- | --- | --- | --- | --- | --- | --- | --- | --- |
| Group 1 | rs10008637 | C | T | 0.022 | -0.004 | 0.462 | 0.401 | 4 | 77414144 | 42457 | 0.681 | 0.010 | 3.90E-08 | 115078 | 0.004 | 0.462 | 0.000 | 26.661 |
| Group 1 | rs10102717 | T | C | -0.028 | -0.014 | 0.393 | 0.362 | 8 | 19756813 | 42457 | 0.161 | 0.010 | 1.00E-11 | 115078 | 0.004 | 0.393 | 0.000 | 41.776 |
| Group 1 | rs10109207 | T | G | -0.070 | -0.086 | 0.034 | 0.032 | 8 | 126462600 | 42457 | 0.050 | 0.044 | 3.10E-09 | 115078 | 0.012 | 0.034 | 0.000 | 36.570 |
| Group 1 | rs1021435 | G | A | -0.024 | -0.009 | 0.562 | 0.558 | 8 | 19577267 | 42457 | 0.333 | 0.010 | 4.60E-09 | 115078 | 0.004 | 0.438 | 0.000 | 33.137 |
| Group 1 | rs10455872 | G | A | -0.101 | 0.319 | 0.079 | 0.055 | 6 | 161010118 | 42457 | 0.000 | 0.024 | 1.00E-42 | 115078 | 0.008 | 0.079 | 0.001 | 170.982 |
| Group 1 | rs10458569 | A | C | -0.072 | -0.022 | 0.041 | 0.054 | 1 | 62997601 | 42457 | 0.275 | 0.020 | 7.20E-13 | 115078 | 0.010 | 0.041 | 0.000 | 46.820 |
| Group 1 | rs1052248 | A | T | 0.027 | 0.007 | 0.258 | 0.308 | 6 | 31556581 | 42457 | 0.529 | 0.010 | 9.70E-09 | 115078 | 0.005 | 0.258 | 0.000 | 32.030 |
| Group 1 | rs1077835 | G | A | 0.203 | 0.037 | 0.220 | 0.245 | 15 | 58723426 | 42457 | 0.001 | 0.011 | 1.00E-200 | 115078 | 0.005 | 0.220 | 0.014 | 1644.838 |
| Group 1 | rs10807083 | A | G | 0.025 | 0.033 | 0.332 | 0.347 | 6 | 31195941 | 42457 | 0.004 | 0.011 | 8.50E-10 | 115078 | 0.004 | 0.332 | 0.000 | 33.051 |
| Group 1 | rs10892004 | T | C | 0.051 | 0.040 | 0.129 | 0.143 | 11 | 116514417 | 42457 | 0.004 | 0.014 | 2.70E-17 | 115078 | 0.006 | 0.129 | 0.001 | 66.370 |
| Group 1 | rs111297471 | C | A | -0.025 | -0.003 | 0.320 | 0.344 | 4 | 88230501 | 42457 | 0.748 | 0.010 | 1.40E-08 | 115078 | 0.004 | 0.320 | 0.000 | 30.419 |
| Group 1 | rs11208050 | C | T | 0.040 | 0.023 | 0.410 | 0.420 | 1 | 63336271 | 42457 | 0.014 | 0.009 | 4.30E-23 | 115078 | 0.004 | 0.410 | 0.001 | 87.761 |
| Group 1 | rs112259268 | A | C | 0.086 | 0.084 | 0.029 | 0.020 | 17 | 41874745 | 42457 | 0.051 | 0.043 | 5.00E-14 | 115078 | 0.012 | 0.029 | 0.000 | 46.810 |
| Group 1 | rs1128249 | T | G | -0.038 | -0.024 | 0.392 | 0.370 | 2 | 165528624 | 42457 | 0.019 | 0.010 | 4.00E-20 | 115078 | 0.004 | 0.392 | 0.001 | 78.131 |
| Group 1 | rs113629348 | T | C | 0.089 | 0.006 | 0.027 | 0.017 | 15 | 58545668 | 42457 | 0.897 | 0.046 | 1.10E-12 | 115078 | 0.013 | 0.027 | 0.000 | 47.852 |
| Group 1 | rs1145210 | T | C | -0.045 | -0.005 | 0.319 | 0.298 | 11 | 116559002 | 42457 | 0.637 | 0.010 | 3.30E-25 | 115078 | 0.004 | 0.319 | 0.001 | 99.862 |
| Group 1 | rs115847023 | C | T | -0.123 | 0.013 | 0.007 | 0.015 | 4 | 185209882 | 42457 | 0.752 | 0.041 | 4.20E-08 | 115078 | 0.024 | 0.007 | 0.000 | 25.673 |
| Group 1 | rs116316096 | A | G | -0.091 | -0.002 | 0.029 | 0.031 | 1 | 62886595 | 42457 | 0.945 | 0.032 | 3.50E-13 | 115078 | 0.013 | 0.029 | 0.000 | 53.053 |
| Group 1 | rs11662059 | A | G | 0.033 | -0.009 | 0.313 | 0.297 | 18 | 47155917 | 42457 | 0.378 | 0.011 | 2.00E-14 | 115078 | 0.004 | 0.313 | 0.000 | 53.965 |
| Group 1 | rs11668738 | G | T | -0.073 | 0.027 | 0.835 | 0.712 | 19 | 45347561 | 42457 | 0.035 | 0.013 | 9.10E-41 | 115078 | 0.006 | 0.165 | 0.001 | 170.903 |
| Group 1 | rs11668861 | T | G | -0.035 | -0.004 | 0.458 | 0.502 | 19 | 45380970 | 42457 | 0.667 | 0.010 | 1.00E-18 | 115078 | 0.004 | 0.458 | 0.001 | 71.482 |
| Group 1 | rs116843064 | A | G | -0.227 | -0.141 | 0.020 | 0.025 | 19 | 8429323 | 42457 | 0.001 | 0.043 | 7.50E-57 | 115078 | 0.015 | 0.020 | 0.002 | 231.110 |
| Group 1 | rs116886525 | T | C | 0.139 | 0.027 | 0.009 | 0.012 | 11 | 116671391 | 42457 | 0.577 | 0.048 | 2.10E-10 | 115078 | 0.021 | 0.009 | 0.000 | 41.602 |
| Group 1 | rs116974927 | C | G | -0.167 | -0.008 | 0.012 | 0.012 | 8 | 19951598 | 42457 | 0.895 | 0.064 | 4.30E-19 | 115078 | 0.019 | 0.012 | 0.001 | 78.334 |
| Group 1 | rs117261169 | T | C | 0.205 | -0.145 | 0.017 | 0.015 | 19 | 45491032 | 42457 | 0.002 | 0.047 | 1.30E-37 | 115078 | 0.016 | 0.017 | 0.001 | 156.858 |
| Group 1 | rs117597286 | C | T | -0.169 | -0.018 | 0.008 | 0.009 | 15 | 58587369 | 42457 | 0.785 | 0.068 | 5.90E-14 | 115078 | 0.023 | 0.008 | 0.000 | 51.793 |
| Group 1 | rs117602337 | T | C | 0.064 | -0.034 | 0.058 | 0.047 | 19 | 45693752 | 42457 | 0.215 | 0.027 | 9.10E-14 | 115078 | 0.009 | 0.058 | 0.000 | 52.047 |
| Group 1 | rs117749052 | C | T | 0.160 | 0.095 | 0.026 | 0.022 | 15 | 58749309 | 42457 | 0.024 | 0.042 | 2.30E-35 | 115078 | 0.013 | 0.026 | 0.001 | 147.026 |
| Group 1 | rs11781692 | A | C | 0.107 | 0.028 | 0.015 | 0.020 | 8 | 19848117 | 42457 | 0.460 | 0.037 | 4.60E-10 | 115078 | 0.017 | 0.015 | 0.000 | 38.375 |
| Group 1 | rs11789603 | T | C | 0.035 | 0.021 | 0.109 | 0.111 | 9 | 107647019 | 42457 | 0.156 | 0.015 | 1.50E-08 | 115078 | 0.007 | 0.109 | 0.000 | 27.213 |
| Group 1 | rs117901517 | C | T | -0.078 | 0.007 | 0.089 | 0.056 | 15 | 58678869 | 42457 | 0.814 | 0.030 | 1.50E-23 | 115078 | 0.008 | 0.089 | 0.001 | 113.604 |
| Group 1 | rs118078695 | A | G | 0.219 | 0.058 | 0.014 | 0.012 | 15 | 58686409 | 42457 | 0.394 | 0.068 | 4.40E-33 | 115078 | 0.019 | 0.014 | 0.001 | 150.873 |
| Group 1 | rs11828763 | G | A | 0.145 | 0.014 | 0.023 | 0.061 | 11 | 116559663 | 42457 | 0.471 | 0.019 | 1.00E-27 | 115078 | 0.014 | 0.023 | 0.001 | 109.863 |
| Group 1 | rs11853431 | A | G | 0.133 | 0.000 | 0.032 | 0.021 | 15 | 58635891 | 42457 | 0.997 | 0.042 | 5.50E-30 | 115078 | 0.012 | 0.032 | 0.001 | 124.037 |
| Group 1 | rs11899121 | C | G | -0.031 | -0.015 | 0.474 | 0.498 | 2 | 20367973 | 42457 | 0.106 | 0.009 | 3.80E-15 | 115078 | 0.004 | 0.474 | 0.000 | 55.865 |
| Group 1 | rs1198433 | G | A | 0.030 | 0.011 | 0.864 | 0.805 | 1 | 23756695 | 42457 | 0.366 | 0.012 | 4.50E-08 | 115078 | 0.006 | 0.136 | 0.000 | 25.071 |
| Group 1 | rs12221682 | G | C | 0.111 | 0.004 | 0.082 | 0.112 | 11 | 116578217 | 42457 | 0.803 | 0.015 | 4.80E-52 | 115078 | 0.007 | 0.082 | 0.002 | 213.405 |
| Group 1 | rs12263369 | T | C | 0.022 | 0.024 | 0.592 | 0.522 | 10 | 94823343 | 42457 | 0.015 | 0.010 | 4.50E-08 | 115078 | 0.004 | 0.408 | 0.000 | 25.856 |
| Group 1 | rs1240658 | C | T | 0.031 | 0.007 | 0.164 | 0.158 | 11 | 116496360 | 42457 | 0.601 | 0.013 | 2.00E-09 | 115078 | 0.006 | 0.164 | 0.000 | 30.331 |
| Group 1 | rs12601919 | G | A | 0.028 | 0.019 | 0.189 | 0.211 | 17 | 65825374 | 42457 | 0.102 | 0.012 | 4.10E-08 | 115078 | 0.005 | 0.189 | 0.000 | 26.760 |
| Group 1 | rs1260326 | C | T | -0.098 | 0.003 | 0.604 | 0.610 | 2 | 27730940 | 42457 | 0.735 | 0.010 | 2.70E-126 | 115078 | 0.004 | 0.396 | 0.005 | 525.747 |
| Group 1 | rs1268353 | T | C | -0.056 | -0.026 | 0.343 | 0.336 | 11 | 116639692 | 42457 | 0.009 | 0.010 | 1.30E-40 | 115078 | 0.004 | 0.343 | 0.001 | 160.087 |
| Group 1 | rs12720816 | C | T | 0.023 | 0.006 | 0.393 | 0.313 | 2 | 21239667 | 42457 | 0.594 | 0.011 | 1.50E-08 | 115078 | 0.004 | 0.393 | 0.000 | 28.551 |
| Group 1 | rs12721041 | T | C | 0.182 | 0.108 | 0.016 | 0.018 | 11 | 116693871 | 42457 | 0.020 | 0.046 | 2.80E-30 | 115078 | 0.016 | 0.016 | 0.001 | 117.756 |
| Group 1 | rs1275505 | A | G | 0.042 | -0.011 | 0.901 | 0.771 | 2 | 27416567 | 42457 | 0.463 | 0.015 | 3.40E-10 | 115078 | 0.007 | 0.099 | 0.000 | 36.741 |
| Group 1 | rs12899879 | C | T | 0.086 | -0.003 | 0.111 | 0.149 | 15 | 58591415 | 42457 | 0.811 | 0.013 | 8.40E-42 | 115078 | 0.007 | 0.111 | 0.001 | 167.258 |
| Group 1 | rs12907378 | A | G | -0.083 | -0.032 | 0.104 | 0.108 | 15 | 58654430 | 42457 | 0.034 | 0.015 | 1.30E-36 | 115078 | 0.007 | 0.104 | 0.001 | 147.575 |
| Group 1 | rs13108218 | G | A | -0.028 | -0.010 | 0.615 | 0.585 | 4 | 3443931 | 42457 | 0.322 | 0.010 | 2.90E-12 | 115078 | 0.004 | 0.385 | 0.000 | 42.790 |
| Group 1 | rs13263508 | T | G | -0.055 | -0.030 | 0.546 | 0.508 | 8 | 19942181 | 42457 | 0.003 | 0.010 | 8.70E-43 | 115078 | 0.004 | 0.454 | 0.002 | 173.768 |
| Group 1 | rs13280055 | A | G | 0.042 | -0.012 | 0.134 | 0.108 | 8 | 11522353 | 42457 | 0.495 | 0.018 | 2.20E-12 | 115078 | 0.006 | 0.134 | 0.000 | 47.475 |
| Group 1 | rs13408252 | A | C | -0.032 | 0.014 | 0.250 | 0.271 | 2 | 27467943 | 42457 | 0.200 | 0.011 | 5.90E-12 | 115078 | 0.005 | 0.250 | 0.000 | 43.854 |
| Group 1 | rs140526322 | T | G | 0.109 | 0.025 | 0.016 | 0.016 | 11 | 116429607 | 42457 | 0.551 | 0.042 | 4.20E-12 | 115078 | 0.016 | 0.016 | 0.000 | 42.963 |
| Group 1 | rs140707095 | G | A | 0.096 | 0.038 | 0.016 | 0.010 | 11 | 116990442 | 42457 | 0.535 | 0.061 | 7.60E-09 | 115078 | 0.017 | 0.016 | 0.000 | 33.195 |
| Group 1 | rs141414463 | T | C | 0.188 | 0.095 | 0.024 | 0.024 | 11 | 116598315 | 42457 | 0.004 | 0.033 | 1.70E-47 | 115078 | 0.013 | 0.024 | 0.002 | 190.529 |
| Group 1 | rs141936239 | C | T | -0.061 | -0.029 | 0.109 | 0.098 | 11 | 117005627 | 42457 | 0.079 | 0.017 | 6.30E-17 | 115078 | 0.007 | 0.109 | 0.001 | 83.323 |
| Group 1 | rs142187704 | C | T | 0.137 | -0.047 | 0.009 | 0.012 | 15 | 58611016 | 42457 | 0.441 | 0.061 | 3.40E-10 | 115078 | 0.023 | 0.009 | 0.000 | 37.703 |
| Group 1 | rs145521244 | A | G | -0.083 | 0.026 | 0.034 | 0.024 | 15 | 58679447 | 42457 | 0.514 | 0.040 | 8.70E-12 | 115078 | 0.012 | 0.034 | 0.000 | 51.742 |
| Group 1 | rs147146149 | G | C | -0.116 | 0.019 | 0.013 | 0.014 | 15 | 58743495 | 42457 | 0.700 | 0.049 | 2.80E-10 | 115078 | 0.018 | 0.013 | 0.000 | 40.756 |
| Group 1 | rs147711004 | A | G | -0.079 | 0.093 | 0.036 | 0.030 | 19 | 45337918 | 42457 | 0.008 | 0.035 | 4.50E-13 | 115078 | 0.011 | 0.036 | 0.000 | 50.515 |
| Group 1 | rs1495741 | A | G | -0.054 | -0.030 | 0.779 | 0.737 | 8 | 18272881 | 42457 | 0.007 | 0.011 | 1.00E-29 | 115078 | 0.005 | 0.221 | 0.001 | 114.140 |
| Group 1 | rs150844304 | C | A | 0.141 | 0.011 | 0.026 | 0.027 | 15 | 43726625 | 42457 | 0.730 | 0.031 | 1.40E-28 | 115078 | 0.013 | 0.026 | 0.001 | 113.801 |
| Group 1 | rs151330717 | A | G | 0.119 | -0.099 | 0.020 | 0.020 | 19 | 45196964 | 42457 | 0.033 | 0.046 | 3.70E-17 | 115078 | 0.015 | 0.020 | 0.001 | 62.014 |
| Group 1 | rs1515098 | T | C | 0.031 | 0.043 | 0.681 | 0.680 | 2 | 227073854 | 42457 | 0.000 | 0.011 | 9.00E-13 | 115078 | 0.004 | 0.319 | 0.000 | 48.908 |
| Group 1 | rs1601935 | T | G | -0.165 | -0.009 | 0.655 | 0.640 | 15 | 58671765 | 42457 | 0.357 | 0.010 | 1.00E-200 | 115078 | 0.004 | 0.345 | 0.012 | 1437.065 |
| Group 1 | rs16842 | C | T | 0.029 | 0.022 | 0.286 | 0.298 | 8 | 19968929 | 42457 | 0.032 | 0.010 | 3.00E-11 | 115078 | 0.005 | 0.286 | 0.000 | 39.516 |
| Group 1 | rs16940147 | A | G | 0.075 | 0.007 | 0.053 | 0.070 | 15 | 58676119 | 42457 | 0.678 | 0.018 | 8.90E-16 | 115078 | 0.009 | 0.053 | 0.001 | 64.528 |
| Group 1 | rs16940262 | C | T | 0.040 | 0.012 | 0.500 | 0.494 | 15 | 58717428 | 42457 | 0.291 | 0.011 | 2.20E-18 | 115078 | 0.005 | 0.500 | 0.001 | 93.001 |
| Group 1 | rs17240566 | A | G | -0.027 | -0.009 | 0.582 | 0.548 | 15 | 58448287 | 42457 | 0.320 | 0.009 | 2.20E-11 | 115078 | 0.004 | 0.418 | 0.000 | 39.369 |
| Group 1 | rs17269250 | G | T | 0.175 | 0.108 | 0.016 | 0.012 | 15 | 58725986 | 42457 | 0.057 | 0.057 | 6.50E-29 | 115078 | 0.016 | 0.016 | 0.001 | 112.428 |
| Group 1 | rs17301746 | T | C | 0.175 | 0.060 | 0.014 | 0.040 | 15 | 58731395 | 42457 | 0.013 | 0.024 | 4.60E-25 | 115078 | 0.017 | 0.014 | 0.001 | 97.045 |
| Group 1 | rs17388017 | T | A | -0.084 | -0.022 | 0.029 | 0.025 | 1 | 63132397 | 42457 | 0.490 | 0.031 | 1.30E-11 | 115078 | 0.012 | 0.029 | 0.000 | 45.861 |
| Group 1 | rs174580 | G | A | 0.047 | -0.021 | 0.352 | 0.327 | 11 | 61606642 | 42457 | 0.039 | 0.010 | 2.20E-28 | 115078 | 0.004 | 0.352 | 0.001 | 114.594 |
| Group 1 | rs17657001 | G | C | 0.067 | -0.025 | 0.044 | 0.043 | 18 | 47224182 | 42457 | 0.323 | 0.025 | 4.40E-11 | 115078 | 0.010 | 0.044 | 0.000 | 42.913 |
| Group 1 | rs186696265 | T | C | -0.211 | 0.550 | 0.014 | 0.013 | 6 | 161111700 | 42457 | 0.000 | 0.048 | 8.50E-38 | 115078 | 0.017 | 0.014 | 0.001 | 145.323 |
| Group 1 | rs186782888 | A | G | 0.149 | 0.085 | 0.013 | 0.013 | 11 | 117086701 | 42457 | 0.124 | 0.055 | 2.30E-15 | 115078 | 0.019 | 0.013 | 0.001 | 64.006 |
| Group 1 | rs187582489 | C | T | 0.029 | -0.006 | 0.513 | 0.462 | 6 | 32539553 | 42457 | 0.631 | 0.013 | 3.60E-10 | 115078 | 0.005 | 0.487 | 0.000 | 46.790 |
| Group 1 | rs187929675 | T | C | -0.122 | 0.111 | 0.013 | 0.014 | 11 | 117111368 | 42457 | 0.073 | 0.062 | 7.40E-11 | 115078 | 0.018 | 0.013 | 0.000 | 44.320 |
| Group 1 | rs188099946 | T | C | 0.130 | -0.134 | 0.025 | 0.019 | 19 | 45189605 | 42457 | 0.000 | 0.038 | 3.70E-23 | 115078 | 0.013 | 0.025 | 0.001 | 94.718 |
| Group 1 | rs1883711 | C | G | 0.073 | 0.134 | 0.031 | 0.035 | 20 | 39179822 | 42457 | 0.000 | 0.035 | 9.00E-11 | 115078 | 0.012 | 0.031 | 0.000 | 37.293 |
| Group 1 | rs190118115 | T | C | 0.155 | 0.080 | 0.019 | 0.022 | 8 | 19793581 | 42457 | 0.051 | 0.041 | 4.20E-26 | 115078 | 0.015 | 0.019 | 0.001 | 101.695 |
| Group 1 | rs1998045 | T | C | -0.046 | 0.054 | 0.158 | 0.141 | 6 | 161097821 | 42457 | 0.000 | 0.014 | 2.60E-18 | 115078 | 0.006 | 0.158 | 0.001 | 65.396 |
| Group 1 | rs2035403 | G | A | 0.033 | 0.009 | 0.398 | 0.360 | 4 | 88018991 | 42457 | 0.383 | 0.010 | 4.30E-16 | 115078 | 0.004 | 0.398 | 0.001 | 59.263 |
| Group 1 | rs2035816 | G | A | -0.042 | -0.064 | 0.084 | 0.075 | 4 | 100508556 | 42457 | 0.000 | 0.018 | 1.30E-09 | 115078 | 0.007 | 0.084 | 0.000 | 32.015 |
| Group 1 | rs204480 | T | C | 0.031 | -0.030 | 0.251 | 0.245 | 19 | 45477111 | 42457 | 0.006 | 0.011 | 1.80E-11 | 115078 | 0.005 | 0.251 | 0.000 | 42.059 |
| Group 1 | rs2119693 | T | G | 0.055 | 0.036 | 0.857 | 0.840 | 8 | 19720395 | 42457 | 0.012 | 0.015 | 6.10E-21 | 115078 | 0.006 | 0.143 | 0.001 | 86.685 |
| Group 1 | rs2156552 | T | A | 0.059 | -0.026 | 0.822 | 0.812 | 18 | 47181668 | 42457 | 0.044 | 0.013 | 1.40E-28 | 115078 | 0.005 | 0.178 | 0.001 | 116.651 |
| Group 1 | rs2228607 | G | A | -0.026 | -0.005 | 0.559 | 0.533 | 7 | 73122923 | 42457 | 0.611 | 0.010 | 2.10E-10 | 115078 | 0.004 | 0.441 | 0.000 | 39.649 |
| Group 1 | rs2356888 | C | G | 0.039 | -0.038 | 0.109 | 0.108 | 19 | 44927688 | 42457 | 0.012 | 0.015 | 2.90E-09 | 115078 | 0.007 | 0.109 | 0.000 | 33.560 |
| Group 1 | rs2409836 | G | T | -0.035 | 0.004 | 0.547 | 0.580 | 8 | 11689228 | 42457 | 0.721 | 0.010 | 6.30E-17 | 115078 | 0.004 | 0.453 | 0.001 | 70.147 |
| Group 1 | rs2414550 | G | C | -0.036 | -0.031 | 0.858 | 0.816 | 15 | 58500757 | 42457 | 0.020 | 0.013 | 1.00E-09 | 115078 | 0.006 | 0.142 | 0.000 | 35.738 |
| Group 1 | rs2425737 | A | G | 0.026 | 0.001 | 0.261 | 0.059 | 20 | 44274875 | 42457 | 0.974 | 0.028 | 4.10E-08 | 115078 | 0.005 | 0.261 | 0.000 | 29.541 |
| Group 1 | rs2524137 | T | C | 0.035 | 0.020 | 0.696 | 0.696 | 6 | 31264582 | 42457 | 0.100 | 0.012 | 2.00E-15 | 115078 | 0.004 | 0.304 | 0.001 | 60.519 |
| Group 1 | rs2596502 | G | A | 0.024 | -0.001 | 0.303 | 0.292 | 6 | 31321032 | 42457 | 0.944 | 0.014 | 2.50E-08 | 115078 | 0.004 | 0.303 | 0.000 | 27.396 |
| Group 1 | rs261264 | T | C | 0.025 | 0.006 | 0.527 | 0.513 | 15 | 58614934 | 42457 | 0.526 | 0.009 | 1.80E-09 | 115078 | 0.004 | 0.473 | 0.000 | 35.882 |
| Group 1 | rs2740488 | C | A | -0.037 | -0.018 | 0.265 | 0.251 | 9 | 107661742 | 42457 | 0.100 | 0.011 | 1.00E-15 | 115078 | 0.005 | 0.265 | 0.001 | 62.059 |
| Group 1 | rs2800708 | T | C | 0.022 | 0.012 | 0.530 | 0.528 | 6 | 127437617 | 42457 | 0.199 | 0.009 | 3.70E-08 | 115078 | 0.004 | 0.470 | 0.000 | 27.788 |
| Group 1 | rs283 | T | C | 0.033 | 0.029 | 0.199 | 0.204 | 8 | 19815098 | 42457 | 0.022 | 0.013 | 8.40E-11 | 115078 | 0.005 | 0.199 | 0.000 | 41.054 |
| Group 1 | rs28383314 | C | T | 0.040 | 0.015 | 0.623 | 0.589 | 6 | 32587213 | 42457 | 0.199 | 0.011 | 1.00E-21 | 115078 | 0.004 | 0.377 | 0.001 | 85.276 |
| Group 1 | rs28399657 | G | A | 0.132 | -0.105 | 0.033 | 0.028 | 19 | 45318351 | 42457 | 0.004 | 0.037 | 1.60E-31 | 115078 | 0.011 | 0.033 | 0.001 | 128.187 |
| Group 1 | rs28550053 | G | A | -0.041 | -0.027 | 0.174 | 0.181 | 8 | 19934305 | 42457 | 0.042 | 0.013 | 5.60E-14 | 115078 | 0.005 | 0.174 | 0.000 | 55.264 |
| Group 1 | rs28601761 | G | C | -0.087 | -0.048 | 0.420 | 0.386 | 8 | 126500031 | 42457 | 0.000 | 0.010 | 1.20E-99 | 115078 | 0.004 | 0.420 | 0.004 | 423.624 |
| Group 1 | rs28607776 | G | A | -0.134 | -0.037 | 0.131 | 0.101 | 15 | 58575005 | 42457 | 0.028 | 0.017 | 2.40E-110 | 115078 | 0.006 | 0.131 | 0.004 | 473.744 |
| Group 1 | rs28631719 | G | A | 0.027 | 0.007 | 0.520 | 0.738 | 6 | 32625409 | 42457 | 0.693 | 0.017 | 2.40E-09 | 115078 | 0.005 | 0.480 | 0.000 | 40.706 |
| Group 1 | rs28752924 | C | T | 0.028 | 0.020 | 0.446 | 0.492 | 6 | 31303922 | 42457 | 0.103 | 0.012 | 1.50E-11 | 115078 | 0.004 | 0.446 | 0.000 | 44.944 |
| Group 1 | rs28917234 | A | G | 0.123 | -0.002 | 0.033 | 0.051 | 11 | 117186506 | 42457 | 0.933 | 0.025 | 1.10E-27 | 115078 | 0.012 | 0.033 | 0.001 | 112.419 |
| Group 1 | rs289744 | T | G | 0.025 | 0.013 | 0.695 | 0.669 | 16 | 57018102 | 42457 | 0.205 | 0.010 | 1.60E-09 | 115078 | 0.004 | 0.305 | 0.000 | 30.730 |
| Group 1 | rs2925979 | C | T | -0.025 | -0.016 | 0.699 | 0.681 | 16 | 81534790 | 42457 | 0.112 | 0.010 | 4.10E-09 | 115078 | 0.004 | 0.301 | 0.000 | 30.779 |
| Group 1 | rs2927481 | A | C | -0.037 | 0.008 | 0.324 | 0.308 | 19 | 45337249 | 42457 | 0.439 | 0.010 | 1.80E-18 | 115078 | 0.004 | 0.324 | 0.001 | 69.137 |
| Group 1 | rs293435 | T | C | 0.026 | 0.019 | 0.289 | 0.327 | 4 | 69588052 | 42457 | 0.093 | 0.011 | 2.30E-08 | 115078 | 0.005 | 0.289 | 0.000 | 31.229 |
| Group 1 | rs2953810 | T | C | -0.023 | 0.012 | 0.595 | 0.588 | 8 | 8872504 | 42457 | 0.228 | 0.010 | 2.30E-08 | 115078 | 0.004 | 0.405 | 0.000 | 28.579 |
| Group 1 | rs2965169 | C | A | 0.033 | -0.023 | 0.388 | 0.429 | 19 | 45251156 | 42457 | 0.021 | 0.010 | 6.80E-15 | 115078 | 0.004 | 0.388 | 0.001 | 58.489 |
| Group 1 | rs2980874 | A | G | -0.033 | -0.010 | 0.388 | 0.336 | 8 | 126444788 | 42457 | 0.454 | 0.013 | 5.60E-16 | 115078 | 0.004 | 0.388 | 0.001 | 60.024 |
| Group 1 | rs3132454 | G | A | 0.029 | 0.001 | 0.340 | 0.388 | 6 | 31489644 | 42457 | 0.948 | 0.010 | 2.60E-11 | 115078 | 0.004 | 0.340 | 0.000 | 42.150 |
| Group 1 | rs3132631 | T | C | -0.032 | -0.048 | 0.144 | 0.107 | 6 | 30344645 | 42457 | 0.021 | 0.021 | 1.20E-08 | 115078 | 0.006 | 0.144 | 0.000 | 28.902 |
| Group 1 | rs328 | G | C | -0.165 | -0.050 | 0.100 | 0.096 | 8 | 19819724 | 42457 | 0.002 | 0.016 | 9.10E-136 | 115078 | 0.007 | 0.100 | 0.005 | 564.924 |
| Group 1 | rs3289 | C | T | 0.119 | 0.093 | 0.027 | 0.029 | 8 | 19823192 | 42457 | 0.002 | 0.030 | 3.40E-21 | 115078 | 0.013 | 0.027 | 0.001 | 87.340 |
| Group 1 | rs34182956 | C | T | 0.031 | 0.000 | 0.193 | 0.144 | 4 | 88208450 | 42457 | 0.987 | 0.015 | 3.60E-10 | 115078 | 0.005 | 0.193 | 0.000 | 34.160 |
| Group 1 | rs34663616 | A | C | 0.054 | 0.021 | 0.138 | 0.132 | 15 | 58569330 | 42457 | 0.167 | 0.015 | 3.00E-19 | 115078 | 0.006 | 0.138 | 0.001 | 79.792 |
| Group 1 | rs34707604 | C | T | 0.027 | 0.008 | 0.259 | 0.215 | 4 | 69491456 | 42457 | 0.667 | 0.019 | 2.50E-08 | 115078 | 0.005 | 0.259 | 0.000 | 32.781 |
| Group 1 | rs34990794 | T | C | 0.037 | 0.014 | 0.146 | 0.137 | 15 | 58601365 | 42457 | 0.320 | 0.014 | 2.90E-11 | 115078 | 0.006 | 0.146 | 0.000 | 38.840 |
| Group 1 | rs35138338 | T | C | -0.054 | -0.016 | 0.278 | 0.264 | 15 | 58744481 | 42457 | 0.242 | 0.014 | 6.00E-32 | 115078 | 0.005 | 0.278 | 0.001 | 132.577 |
| Group 1 | rs35169799 | T | C | 0.046 | 0.031 | 0.063 | 0.051 | 11 | 64031241 | 42457 | 0.181 | 0.023 | 3.80E-08 | 115078 | 0.008 | 0.063 | 0.000 | 28.495 |
| Group 1 | rs35686293 | A | G | 0.065 | -0.001 | 0.054 | 0.051 | 2 | 27538635 | 42457 | 0.962 | 0.026 | 1.70E-13 | 115078 | 0.009 | 0.054 | 0.000 | 49.556 |
| Group 1 | rs35823804 | A | G | 0.033 | -0.016 | 0.196 | 0.247 | 19 | 45135442 | 42457 | 0.140 | 0.011 | 2.90E-11 | 115078 | 0.005 | 0.196 | 0.000 | 39.419 |
| Group 1 | rs35867424 | A | G | -0.093 | 0.006 | 0.030 | 0.019 | 7 | 72032358 | 42457 | 0.913 | 0.052 | 5.30E-14 | 115078 | 0.012 | 0.030 | 0.000 | 56.998 |
| Group 1 | rs36177368 | G | A | 0.025 | 0.013 | 0.259 | 0.232 | 2 | 21182710 | 42457 | 0.256 | 0.012 | 1.70E-08 | 115078 | 0.005 | 0.259 | 0.000 | 28.654 |
| Group 1 | rs3763432 | T | C | 0.031 | -0.002 | 0.401 | 0.393 | 7 | 72974869 | 42457 | 0.804 | 0.010 | 4.50E-14 | 115078 | 0.004 | 0.401 | 0.000 | 54.006 |
| Group 1 | rs3812316 | G | C | -0.110 | 0.010 | 0.129 | 0.119 | 7 | 73020337 | 42457 | 0.502 | 0.015 | 4.30E-75 | 115078 | 0.006 | 0.129 | 0.003 | 311.673 |
| Group 1 | rs3898938 | T | C | -0.031 | -0.024 | 0.482 | 0.478 | 8 | 19749390 | 42457 | 0.010 | 0.009 | 3.50E-14 | 115078 | 0.004 | 0.482 | 0.000 | 54.880 |
| Group 1 | rs3899015 | T | C | -0.047 | -0.037 | 0.141 | 0.126 | 8 | 19668707 | 42457 | 0.016 | 0.015 | 2.60E-17 | 115078 | 0.006 | 0.141 | 0.001 | 62.406 |
| Group 1 | rs3936511 | G | A | 0.044 | 0.044 | 0.192 | 0.167 | 5 | 55860781 | 42457 | 0.000 | 0.012 | 2.50E-17 | 115078 | 0.005 | 0.192 | 0.001 | 68.029 |
| Group 1 | rs4006564 | C | T | -0.035 | 0.004 | 0.552 | 0.510 | 8 | 126531526 | 42457 | 0.752 | 0.012 | 4.90E-15 | 115078 | 0.005 | 0.448 | 0.001 | 67.896 |
| Group 1 | rs402465 | T | C | -0.072 | -0.009 | 0.793 | 0.747 | 15 | 58705029 | 42457 | 0.461 | 0.012 | 1.40E-48 | 115078 | 0.005 | 0.207 | 0.002 | 198.010 |
| Group 1 | rs40270 | C | A | 0.030 | 0.019 | 0.772 | 0.701 | 5 | 55804552 | 42457 | 0.073 | 0.011 | 1.40E-11 | 115078 | 0.005 | 0.228 | 0.000 | 35.795 |
| Group 1 | rs405509 | G | T | 0.062 | -0.051 | 0.511 | 0.497 | 19 | 45408836 | 42457 | 0.000 | 0.010 | 1.10E-52 | 115078 | 0.004 | 0.489 | 0.002 | 223.932 |
| Group 1 | rs4263041 | G | A | 0.042 | 0.003 | 0.283 | 0.317 | 19 | 45438643 | 42457 | 0.866 | 0.016 | 4.70E-18 | 115078 | 0.005 | 0.283 | 0.001 | 80.551 |
| Group 1 | rs445093 | T | C | 0.048 | 0.003 | 0.384 | 0.336 | 15 | 58644949 | 42457 | 0.745 | 0.010 | 9.60E-32 | 115078 | 0.004 | 0.384 | 0.001 | 126.753 |
| Group 1 | rs449647 | T | A | 0.052 | -0.033 | 0.165 | 0.166 | 19 | 45408564 | 42457 | 0.031 | 0.015 | 8.50E-21 | 115078 | 0.006 | 0.165 | 0.001 | 87.039 |
| Group 1 | rs4635554 | G | T | 0.024 | 0.041 | 0.338 | 0.333 | 2 | 21389659 | 42457 | 0.000 | 0.010 | 6.70E-10 | 115078 | 0.004 | 0.338 | 0.000 | 30.738 |
| Group 1 | rs4665710 | C | A | 0.065 | 0.000 | 0.793 | 0.707 | 2 | 21221035 | 42457 | 0.987 | 0.011 | 2.20E-40 | 115078 | 0.005 | 0.207 | 0.001 | 161.075 |
| Group 1 | rs4666042 | A | G | 0.028 | 0.024 | 0.745 | 0.714 | 2 | 28421461 | 42457 | 0.024 | 0.011 | 3.00E-10 | 115078 | 0.005 | 0.255 | 0.000 | 35.437 |
| Group 1 | rs4704834 | G | A | 0.023 | 0.013 | 0.644 | 0.617 | 5 | 156443066 | 42457 | 0.185 | 0.010 | 2.50E-08 | 115078 | 0.004 | 0.356 | 0.000 | 27.326 |
| Group 1 | rs4722551 | C | T | -0.038 | 0.008 | 0.158 | 0.147 | 7 | 25991826 | 42457 | 0.559 | 0.013 | 5.70E-13 | 115078 | 0.006 | 0.158 | 0.000 | 43.464 |
| Group 1 | rs4774292 | C | T | 0.024 | 0.012 | 0.582 | 0.595 | 15 | 58542197 | 42457 | 0.209 | 0.010 | 8.50E-09 | 115078 | 0.004 | 0.418 | 0.000 | 33.496 |
| Group 1 | rs4775075 | G | A | 0.043 | 0.024 | 0.083 | 0.105 | 15 | 58837764 | 42457 | 0.136 | 0.016 | 1.40E-09 | 115078 | 0.007 | 0.083 | 0.000 | 32.661 |
| Group 1 | rs4775085 | A | G | -0.056 | -0.024 | 0.051 | 0.041 | 15 | 58949991 | 42457 | 0.347 | 0.026 | 1.40E-09 | 115078 | 0.009 | 0.051 | 0.000 | 35.308 |
| Group 1 | rs4803791 | A | G | 0.040 | -0.019 | 0.226 | 0.269 | 19 | 45523583 | 42457 | 0.145 | 0.013 | 2.60E-16 | 115078 | 0.005 | 0.226 | 0.001 | 64.776 |
| Group 1 | rs4804311 | G | A | -0.045 | -0.063 | 0.083 | 0.070 | 19 | 8615589 | 42457 | 0.002 | 0.021 | 1.80E-08 | 115078 | 0.007 | 0.083 | 0.000 | 34.880 |
| Group 1 | rs4921893 | C | A | 0.022 | 0.004 | 0.440 | 0.482 | 8 | 18194218 | 42457 | 0.674 | 0.010 | 1.60E-08 | 115078 | 0.004 | 0.440 | 0.000 | 27.188 |
| Group 1 | rs5090 | G | C | -0.050 | -0.025 | 0.062 | 0.050 | 11 | 116694055 | 42457 | 0.319 | 0.025 | 7.90E-09 | 115078 | 0.009 | 0.062 | 0.000 | 33.005 |
| Group 1 | rs523549 | G | A | 0.039 | 0.016 | 0.585 | 0.641 | 11 | 116511028 | 42457 | 0.153 | 0.011 | 1.90E-20 | 115078 | 0.004 | 0.415 | 0.001 | 82.928 |
| Group 1 | rs55687425 | C | T | -0.035 | 0.007 | 0.154 | 0.180 | 5 | 55866886 | 42457 | 0.596 | 0.013 | 1.60E-09 | 115078 | 0.006 | 0.154 | 0.000 | 37.197 |
| Group 1 | rs55807911 | C | T | 0.039 | 0.005 | 0.198 | 0.184 | 2 | 28421473 | 42457 | 0.695 | 0.013 | 9.10E-15 | 115078 | 0.005 | 0.198 | 0.000 | 54.309 |
| Group 1 | rs55995747 | G | C | -0.039 | -0.021 | 0.147 | 0.122 | 15 | 58789530 | 42457 | 0.178 | 0.016 | 7.60E-12 | 115078 | 0.006 | 0.147 | 0.000 | 43.871 |
| Group 1 | rs56001710 | T | A | -0.031 | 0.000 | 0.581 | 0.511 | 7 | 25983400 | 42457 | 0.967 | 0.010 | 4.30E-14 | 115078 | 0.004 | 0.419 | 0.000 | 52.780 |
| Group 1 | rs56156922 | C | T | -0.057 | -0.030 | 0.324 | 0.306 | 16 | 56987369 | 42457 | 0.003 | 0.010 | 2.40E-41 | 115078 | 0.004 | 0.324 | 0.001 | 164.061 |
| Group 1 | rs56819515 | A | T | 0.039 | -0.020 | 0.127 | 0.137 | 3 | 170712913 | 42457 | 0.139 | 0.014 | 3.50E-10 | 115078 | 0.006 | 0.127 | 0.000 | 37.902 |
| Group 1 | rs571848809 | A | G | 0.065 | -0.066 | 0.106 | 0.090 | 6 | 161005389 | 42457 | 0.000 | 0.018 | 4.90E-25 | 115078 | 0.007 | 0.106 | 0.001 | 92.378 |
| Group 1 | rs579890 | G | A | -0.105 | -0.012 | 0.898 | 0.821 | 11 | 116798963 | 42457 | 0.348 | 0.012 | 2.30E-57 | 115078 | 0.007 | 0.102 | 0.002 | 235.035 |
| Group 1 | rs595137 | T | C | -0.145 | 0.004 | 0.978 | 0.932 | 11 | 116771356 | 42457 | 0.878 | 0.027 | 4.60E-26 | 115078 | 0.014 | 0.022 | 0.001 | 103.754 |
| Group 1 | rs59752567 | T | G | 0.075 | 0.017 | 0.086 | 0.100 | 15 | 58791416 | 42457 | 0.320 | 0.017 | 8.50E-25 | 115078 | 0.007 | 0.086 | 0.001 | 102.771 |
| Group 1 | rs59999923 | C | G | 0.190 | -0.057 | 0.009 | 0.020 | 15 | 58734397 | 42457 | 0.096 | 0.034 | 1.30E-15 | 115078 | 0.023 | 0.009 | 0.001 | 72.519 |
| Group 1 | rs6124249 | C | T | -0.025 | -0.027 | 0.315 | 0.323 | 20 | 39175029 | 42457 | 0.006 | 0.010 | 3.20E-09 | 115078 | 0.004 | 0.315 | 0.000 | 30.159 |
| Group 1 | rs61362984 | G | A | -0.025 | 0.001 | 0.379 | 0.326 | 11 | 116450463 | 42457 | 0.950 | 0.011 | 1.70E-09 | 115078 | 0.004 | 0.379 | 0.000 | 33.977 |
| Group 1 | rs61904855 | A | C | 0.067 | 0.070 | 0.028 | 0.017 | 11 | 116476668 | 42457 | 0.145 | 0.048 | 1.60E-09 | 115078 | 0.012 | 0.028 | 0.000 | 28.293 |
| Group 1 | rs62000868 | G | C | 0.126 | 0.003 | 0.281 | 0.254 | 15 | 58705980 | 42457 | 0.817 | 0.012 | 8.99E-174 | 115078 | 0.005 | 0.281 | 0.006 | 740.263 |
| Group 1 | rs62001848 | A | C | 0.099 | 0.004 | 0.023 | 0.024 | 15 | 58786045 | 42457 | 0.926 | 0.039 | 6.50E-13 | 115078 | 0.014 | 0.023 | 0.000 | 51.822 |
| Group 1 | rs62020698 | T | C | 0.042 | -0.004 | 0.092 | 0.070 | 15 | 43237414 | 42457 | 0.864 | 0.024 | 5.10E-10 | 115078 | 0.007 | 0.092 | 0.000 | 34.568 |
| Group 1 | rs62117161 | G | A | 0.084 | -0.031 | 0.088 | 0.091 | 19 | 45233385 | 42457 | 0.059 | 0.016 | 2.20E-32 | 115078 | 0.007 | 0.088 | 0.001 | 130.547 |
| Group 1 | rs62138973 | G | C | 0.059 | -0.008 | 0.094 | 0.082 | 2 | 27847642 | 42457 | 0.691 | 0.019 | 3.50E-18 | 115078 | 0.007 | 0.094 | 0.001 | 68.185 |
| Group 1 | rs62173895 | C | G | 0.026 | 0.012 | 0.670 | 0.616 | 2 | 165477903 | 42457 | 0.243 | 0.010 | 3.80E-10 | 115078 | 0.004 | 0.330 | 0.000 | 35.195 |
| Group 1 | rs632057 | G | T | -0.033 | -0.012 | 0.628 | 0.600 | 6 | 139834012 | 42457 | 0.197 | 0.010 | 1.10E-14 | 115078 | 0.004 | 0.372 | 0.001 | 57.756 |
| Group 1 | rs6493982 | T | C | -0.023 | 0.008 | 0.581 | 0.538 | 15 | 58374707 | 42457 | 0.406 | 0.010 | 4.90E-09 | 115078 | 0.004 | 0.419 | 0.000 | 30.375 |
| Group 1 | rs6507945 | C | A | 0.037 | 0.016 | 0.551 | 0.550 | 18 | 47243912 | 42457 | 0.099 | 0.009 | 1.60E-18 | 115078 | 0.004 | 0.449 | 0.001 | 79.586 |
| Group 1 | rs6587973 | G | A | -0.044 | -0.023 | 0.279 | 0.297 | 1 | 62840786 | 42457 | 0.043 | 0.011 | 1.90E-23 | 115078 | 0.005 | 0.279 | 0.001 | 91.459 |
| Group 1 | rs6606725 | A | C | 0.023 | 0.004 | 0.440 | 0.441 | 12 | 109905368 | 42457 | 0.661 | 0.009 | 4.80E-09 | 115078 | 0.004 | 0.440 | 0.000 | 29.255 |
| Group 1 | rs6675401 | T | C | -0.103 | -0.012 | 0.352 | 0.349 | 1 | 63073975 | 42457 | 0.203 | 0.010 | 1.90E-134 | 115078 | 0.004 | 0.352 | 0.005 | 561.377 |
| Group 1 | rs67120644 | C | T | -0.040 | -0.034 | 0.171 | 0.133 | 11 | 116625897 | 42457 | 0.027 | 0.015 | 5.00E-15 | 115078 | 0.005 | 0.171 | 0.000 | 52.820 |
| Group 1 | rs684773 | C | A | 0.035 | 0.034 | 0.767 | 0.765 | 3 | 135956305 | 42457 | 0.003 | 0.011 | 2.10E-14 | 115078 | 0.005 | 0.233 | 0.000 | 50.563 |
| Group 1 | rs688456 | T | G | 0.066 | 0.009 | 0.192 | 0.224 | 11 | 116722551 | 42457 | 0.406 | 0.011 | 1.20E-37 | 115078 | 0.005 | 0.192 | 0.001 | 154.822 |
| Group 1 | rs6938550 | A | G | -0.039 | -0.008 | 0.914 | 0.881 | 6 | 20462138 | 42457 | 0.618 | 0.017 | 1.70E-08 | 115078 | 0.007 | 0.086 | 0.000 | 26.798 |
| Group 1 | rs7005453 | A | G | 0.035 | 0.012 | 0.211 | 0.235 | 8 | 126629700 | 42457 | 0.287 | 0.011 | 9.90E-13 | 115078 | 0.005 | 0.211 | 0.000 | 48.255 |
| Group 1 | rs7012891 | C | T | 0.049 | 0.029 | 0.237 | 0.291 | 8 | 126514676 | 42457 | 0.005 | 0.011 | 1.90E-24 | 115078 | 0.005 | 0.237 | 0.001 | 99.564 |
| Group 1 | rs704 | A | G | -0.025 | 0.025 | 0.476 | 0.484 | 17 | 26694861 | 42457 | 0.008 | 0.009 | 4.00E-10 | 115078 | 0.004 | 0.476 | 0.000 | 34.597 |
| Group 1 | rs71480323 | A | G | -0.034 | -0.003 | 0.122 | 0.109 | 11 | 116771600 | 42457 | 0.850 | 0.016 | 8.80E-09 | 115078 | 0.006 | 0.122 | 0.000 | 28.208 |
| Group 1 | rs7229377 | T | C | 0.031 | -0.014 | 0.201 | 0.234 | 18 | 47148299 | 42457 | 0.229 | 0.011 | 3.90E-10 | 115078 | 0.005 | 0.201 | 0.000 | 35.858 |
| Group 1 | rs72647336 | A | G | 0.074 | 0.044 | 0.057 | 0.059 | 8 | 126445055 | 42457 | 0.083 | 0.025 | 1.40E-14 | 115078 | 0.009 | 0.057 | 0.001 | 67.114 |
| Group 1 | rs72647352 | C | T | 0.035 | 0.011 | 0.167 | 0.127 | 8 | 126452509 | 42457 | 0.478 | 0.016 | 1.10E-10 | 115078 | 0.006 | 0.167 | 0.000 | 38.549 |
| Group 1 | rs72655677 | A | G | -0.072 | -0.042 | 0.052 | 0.051 | 8 | 126484897 | 42457 | 0.140 | 0.028 | 5.40E-14 | 115078 | 0.010 | 0.052 | 0.001 | 59.151 |
| Group 1 | rs72737411 | A | G | -0.036 | 0.023 | 0.121 | 0.107 | 15 | 58264605 | 42457 | 0.183 | 0.017 | 1.40E-09 | 115078 | 0.006 | 0.121 | 0.000 | 32.457 |
| Group 1 | rs72740858 | G | A | 0.145 | 0.006 | 0.023 | 0.025 | 15 | 58705943 | 42457 | 0.889 | 0.041 | 4.40E-26 | 115078 | 0.014 | 0.023 | 0.001 | 109.012 |
| Group 1 | rs72743576 | A | T | -0.104 | 0.009 | 0.016 | 0.036 | 15 | 58454686 | 42457 | 0.716 | 0.024 | 8.70E-11 | 115078 | 0.016 | 0.016 | 0.000 | 40.009 |
| Group 1 | rs72929768 | C | T | -0.048 | -0.016 | 0.811 | 0.774 | 1 | 62882603 | 42457 | 0.163 | 0.011 | 1.20E-21 | 115078 | 0.005 | 0.189 | 0.001 | 81.909 |
| Group 1 | rs73048351 | A | C | 0.127 | -0.048 | 0.010 | 0.012 | 19 | 45160086 | 42457 | 0.465 | 0.065 | 1.60E-09 | 115078 | 0.021 | 0.010 | 0.000 | 37.478 |
| Group 1 | rs73420594 | T | C | -0.073 | -0.016 | 0.079 | 0.073 | 15 | 58741401 | 42457 | 0.399 | 0.018 | 2.30E-22 | 115078 | 0.008 | 0.079 | 0.001 | 89.445 |
| Group 1 | rs737338 | T | C | -0.087 | 0.018 | 0.035 | 0.077 | 19 | 11347657 | 42457 | 0.287 | 0.017 | 8.30E-14 | 115078 | 0.011 | 0.035 | 0.001 | 58.578 |
| Group 1 | rs739320 | C | T | -0.030 | 0.002 | 0.607 | 0.661 | 19 | 49261368 | 42457 | 0.881 | 0.013 | 2.60E-11 | 115078 | 0.004 | 0.393 | 0.000 | 48.772 |
| Group 1 | rs74018729 | A | G | 0.142 | 0.048 | 0.028 | 0.026 | 15 | 58641140 | 42457 | 0.121 | 0.031 | 3.60E-31 | 115078 | 0.012 | 0.028 | 0.001 | 127.768 |
| Group 1 | rs7461833 | A | G | 0.049 | 0.031 | 0.061 | 0.065 | 8 | 126469139 | 42457 | 0.106 | 0.019 | 5.70E-09 | 115078 | 0.009 | 0.061 | 0.000 | 31.526 |
| Group 1 | rs7550306 | C | A | 0.049 | -0.002 | 0.178 | 0.154 | 1 | 62997573 | 42457 | 0.869 | 0.014 | 4.60E-19 | 115078 | 0.005 | 0.178 | 0.001 | 80.142 |
| Group 1 | rs7588926 | T | C | -0.034 | 0.012 | 0.182 | 0.156 | 2 | 27342894 | 42457 | 0.419 | 0.015 | 1.20E-11 | 115078 | 0.005 | 0.182 | 0.000 | 40.151 |
| Group 1 | rs75919952 | T | C | -0.052 | 0.000 | 0.050 | 0.051 | 11 | 116667545 | 42457 | 0.993 | 0.022 | 1.30E-08 | 115078 | 0.009 | 0.050 | 0.000 | 29.111 |
| Group 1 | rs76769796 | G | A | -0.043 | 0.000 | 0.127 | 0.132 | 8 | 19686315 | 42457 | 1.000 | 0.014 | 1.70E-13 | 115078 | 0.006 | 0.127 | 0.000 | 47.014 |
| Group 1 | rs7679 | C | T | 0.055 | -0.026 | 0.186 | 0.170 | 20 | 44576502 | 42457 | 0.035 | 0.012 | 3.10E-28 | 115078 | 0.005 | 0.186 | 0.001 | 104.593 |
| Group 1 | rs76895963 | G | T | -0.093 | 0.000 | 0.021 | 0.023 | 12 | 4384844 | 42457 | 0.999 | 0.047 | 1.10E-08 | 115078 | 0.016 | 0.021 | 0.000 | 40.367 |
| Group 1 | rs76993561 | G | C | 0.059 | 0.052 | 0.038 | 0.039 | 8 | 126499543 | 42457 | 0.042 | 0.026 | 4.30E-08 | 115078 | 0.011 | 0.038 | 0.000 | 29.319 |
| Group 1 | rs77027049 | T | C | 0.069 | -0.021 | 0.066 | 0.070 | 15 | 58716377 | 42457 | 0.413 | 0.026 | 6.00E-17 | 115078 | 0.008 | 0.066 | 0.001 | 67.820 |
| Group 1 | rs77449055 | A | G | -0.072 | 0.034 | 0.041 | 0.040 | 1 | 63342388 | 42457 | 0.213 | 0.027 | 1.50E-11 | 115078 | 0.010 | 0.041 | 0.000 | 46.777 |
| Group 1 | rs77524918 | C | T | -0.070 | -0.016 | 0.046 | 0.031 | 1 | 63042465 | 42457 | 0.611 | 0.031 | 3.00E-14 | 115078 | 0.010 | 0.046 | 0.000 | 49.380 |
| Group 1 | rs77753174 | G | A | 0.040 | -0.020 | 0.171 | 0.146 | 8 | 10732082 | 42457 | 0.165 | 0.015 | 2.30E-13 | 115078 | 0.005 | 0.171 | 0.000 | 53.409 |
| Group 1 | rs77842142 | T | C | -0.202 | -0.124 | 0.005 | 0.012 | 8 | 19902490 | 42457 | 0.071 | 0.069 | 1.30E-10 | 115078 | 0.032 | 0.005 | 0.000 | 47.655 |
| Group 1 | rs77960347 | G | A | 0.203 | -0.102 | 0.013 | 0.011 | 18 | 47109955 | 42457 | 0.045 | 0.051 | 4.80E-32 | 115078 | 0.018 | 0.013 | 0.001 | 123.786 |
| Group 1 | rs7835546 | C | T | -0.028 | -0.001 | 0.597 | 0.573 | 8 | 19615864 | 42457 | 0.932 | 0.010 | 5.20E-12 | 115078 | 0.004 | 0.403 | 0.000 | 42.074 |
| Group 1 | rs78425119 | A | G | 0.048 | 0.016 | 0.068 | 0.057 | 6 | 160501825 | 42457 | 0.468 | 0.023 | 1.40E-10 | 115078 | 0.008 | 0.068 | 0.000 | 33.914 |
| Group 1 | rs78893833 | G | A | 0.119 | 0.026 | 0.023 | 0.018 | 15 | 58688159 | 42457 | 0.607 | 0.050 | 1.40E-19 | 115078 | 0.014 | 0.023 | 0.001 | 72.472 |
| Group 1 | rs78936960 | A | C | -0.046 | 0.007 | 0.090 | 0.059 | 15 | 58717401 | 42457 | 0.831 | 0.033 | 5.40E-11 | 115078 | 0.007 | 0.090 | 0.000 | 39.957 |
| Group 1 | rs79152165 | A | G | 0.055 | 0.061 | 0.044 | 0.035 | 1 | 63092886 | 42457 | 0.045 | 0.030 | 2.80E-08 | 115078 | 0.010 | 0.044 | 0.000 | 28.790 |
| Group 1 | rs79419121 | C | T | 0.112 | -0.003 | 0.026 | 0.016 | 15 | 58708443 | 42457 | 0.950 | 0.047 | 1.40E-18 | 115078 | 0.013 | 0.026 | 0.001 | 72.874 |
| Group 1 | rs79468673 | G | A | 0.062 | -0.015 | 0.035 | 0.038 | 2 | 28254894 | 42457 | 0.589 | 0.027 | 6.90E-09 | 115078 | 0.011 | 0.035 | 0.000 | 30.334 |
| Group 1 | rs79717793 | A | G | -0.032 | 0.003 | 0.154 | 0.136 | 10 | 5262267 | 42457 | 0.833 | 0.014 | 4.60E-09 | 115078 | 0.006 | 0.154 | 0.000 | 30.573 |
| Group 1 | rs7979473 | G | A | -0.031 | -0.034 | 0.613 | 0.564 | 12 | 121420260 | 42457 | 0.000 | 0.009 | 2.20E-13 | 115078 | 0.004 | 0.387 | 0.000 | 53.708 |
| Group 1 | rs799158 | C | T | -0.074 | -0.033 | 0.957 | 0.967 | 7 | 73019074 | 42457 | 0.353 | 0.036 | 1.00E-13 | 115078 | 0.010 | 0.043 | 0.000 | 52.097 |
| Group 1 | rs80289704 | A | G | 0.066 | 0.013 | 0.025 | 0.021 | 15 | 58710653 | 42457 | 0.752 | 0.040 | 3.80E-08 | 115078 | 0.013 | 0.025 | 0.000 | 24.530 |
| Group 1 | rs8042174 | C | T | -0.123 | -0.018 | 0.106 | 0.124 | 15 | 58685970 | 42457 | 0.197 | 0.014 | 4.60E-77 | 115078 | 0.007 | 0.106 | 0.003 | 332.092 |
| Group 1 | rs8045855 | A | T | 0.054 | 0.023 | 0.185 | 0.201 | 16 | 57000696 | 42457 | 0.057 | 0.012 | 6.80E-27 | 115078 | 0.005 | 0.185 | 0.001 | 102.768 |
| Group 1 | rs8107974 | T | A | -0.065 | -0.047 | 0.076 | 0.084 | 19 | 19388500 | 42457 | 0.007 | 0.017 | 4.70E-17 | 115078 | 0.008 | 0.076 | 0.001 | 67.494 |
| Group 1 | rs814573 | T | A | -0.037 | 0.074 | 0.186 | 0.167 | 19 | 45424351 | 42457 | 0.000 | 0.017 | 3.10E-12 | 115078 | 0.005 | 0.186 | 0.000 | 49.053 |
| Group 1 | rs8179210 | G | C | 0.082 | 0.003 | 0.021 | 0.021 | 2 | 27724535 | 42457 | 0.930 | 0.035 | 1.50E-08 | 115078 | 0.014 | 0.021 | 0.000 | 31.193 |
| Group 1 | rs8192701 | T | C | -0.072 | -0.014 | 0.153 | 0.140 | 15 | 58724371 | 42457 | 0.301 | 0.014 | 1.90E-38 | 115078 | 0.006 | 0.153 | 0.001 | 156.283 |
| Group 1 | rs863750 | T | C | 0.022 | 0.028 | 0.607 | 0.589 | 12 | 124505444 | 42457 | 0.003 | 0.009 | 4.20E-08 | 115078 | 0.004 | 0.393 | 0.000 | 26.691 |
| Group 1 | rs9267857 | G | A | 0.026 | -0.015 | 0.413 | 0.188 | 6 | 32196569 | 42457 | 0.334 | 0.016 | 3.70E-10 | 115078 | 0.004 | 0.413 | 0.000 | 36.492 |
| Group 1 | rs9457827 | T | C | -0.124 | 0.046 | 0.018 | 0.111 | 6 | 160503656 | 42457 | 0.002 | 0.015 | 1.00E-14 | 115078 | 0.016 | 0.018 | 0.001 | 63.275 |
| Group 1 | rs964184 | C | G | -0.215 | -0.050 | 0.867 | 0.815 | 11 | 116648917 | 42457 | 0.000 | 0.012 | 1.00E-200 | 115078 | 0.006 | 0.133 | 0.011 | 1238.767 |
| Group 1 | rs9644636 | G | T | 0.032 | 0.026 | 0.278 | 0.268 | 8 | 19824896 | 42457 | 0.018 | 0.011 | 1.90E-12 | 115078 | 0.005 | 0.278 | 0.000 | 48.414 |
| Group 1 | rs9644684 | C | T | -0.029 | -0.004 | 0.231 | 0.274 | 8 | 10430604 | 42457 | 0.722 | 0.012 | 5.90E-09 | 115078 | 0.005 | 0.231 | 0.000 | 33.679 |
| Group 1 | rs998584 | A | C | 0.024 | 0.042 | 0.482 | 0.505 | 6 | 43757896 | 42457 | 0.000 | 0.010 | 5.50E-10 | 115078 | 0.004 | 0.482 | 0.000 | 33.776 |
| Group 2 | rs11204087 | T | C | -0.038 | -0.037 | 0.430 | 0.428 | 8 | 19943027 | 105552 | 0.001 | 0.011 | 3.50E-08 | 37359 | 0.007 | 0.430 | 0.001 | 25.823 |
| Group 2 | rs1168013 | C | G | -0.080 | -0.012 | 0.353 | 0.348 | 1 | 62996838 | 104534 | 0.283 | 0.012 | 7.30E-27 | 37359 | 0.007 | 0.353 | 0.003 | 108.750 |
| Group 2 | rs12478327 | C | A | -0.045 | 0.004 | 0.470 | 0.509 | 2 | 20374249 | 106579 | 0.693 | 0.011 | 2.40E-11 | 37359 | 0.007 | 0.470 | 0.001 | 38.216 |
| Group 2 | rs1260326 | T | C | 0.067 | -0.023 | 0.397 | 0.366 | 2 | 27742603 | 105434 | 0.053 | 0.012 | 6.50E-21 | 37359 | 0.007 | 0.397 | 0.002 | 79.770 |
| Group 2 | rs12900622 | C | T | -0.091 | 0.019 | 0.153 | 0.164 | 15 | 58731455 | 102803 | 0.220 | 0.015 | 9.30E-22 | 37359 | 0.010 | 0.153 | 0.002 | 80.288 |
| Group 2 | rs1373655 | T | G | -0.056 | 0.003 | 0.304 | 0.286 | 15 | 58611859 | 101860 | 0.784 | 0.012 | 1.60E-13 | 37359 | 0.008 | 0.304 | 0.001 | 49.196 |
| Group 2 | rs1532085 | A | G | 0.165 | 0.028 | 0.387 | 0.404 | 15 | 58683366 | 106779 | 0.012 | 0.011 | 4.60E-116 | 37359 | 0.007 | 0.387 | 0.013 | 486.375 |
| Group 2 | rs17321515 | G | A | -0.081 | -0.045 | 0.472 | 0.430 | 8 | 126495818 | 102670 | 0.000 | 0.012 | 2.60E-32 | 37359 | 0.007 | 0.472 | 0.003 | 122.083 |
| Group 2 | rs1800588 | T | C | 0.226 | 0.027 | 0.212 | 0.217 | 15 | 58730498 | 105671 | 0.043 | 0.013 | 6.10E-156 | 37359 | 0.009 | 0.212 | 0.017 | 647.688 |
| Group 2 | rs2575876 | A | G | -0.052 | -0.006 | 0.251 | 0.260 | 9 | 107665739 | 106597 | 0.651 | 0.012 | 2.40E-11 | 37359 | 0.008 | 0.251 | 0.001 | 38.330 |
| Group 2 | rs325 | C | T | -0.125 | -0.066 | 0.102 | 0.107 | 8 | 19819328 | 107133 | 0.016 | 0.027 | 2.00E-27 | 37359 | 0.012 | 0.102 | 0.003 | 106.952 |
| Group 2 | rs442177 | G | T | -0.039 | -0.001 | 0.405 | 0.428 | 4 | 88030261 | 107286 | 0.919 | 0.011 | 2.60E-08 | 37359 | 0.007 | 0.405 | 0.001 | 26.983 |
| Group 2 | rs4775031 | A | C | -0.063 | -0.011 | 0.133 | 0.139 | 15 | 58635583 | 100564 | 0.515 | 0.017 | 9.50E-10 | 37359 | 0.010 | 0.133 | 0.001 | 34.198 |
| Group 2 | rs533617 | C | T | -0.115 | 0.017 | 0.039 | 0.035 | 2 | 21233972 | 100563 | 0.597 | 0.031 | 4.30E-10 | 37359 | 0.018 | 0.039 | 0.001 | 37.071 |
| Group 2 | rs7112513 | A | G | 0.094 | 0.016 | 0.102 | 0.138 | 11 | 117042377 | 107358 | 0.281 | 0.015 | 1.10E-17 | 37359 | 0.011 | 0.102 | 0.002 | 61.145 |
| Group 2 | rs7240405 | A | G | -0.061 | 0.014 | 0.173 | 0.183 | 18 | 47159090 | 106475 | 0.355 | 0.015 | 4.50E-12 | 37359 | 0.009 | 0.173 | 0.001 | 39.761 |
| Group 2 | rs7679 | C | T | -0.059 | -0.008 | 0.183 | 0.203 | 20 | 44576502 | 101011 | 0.574 | 0.014 | 7.00E-11 | 37359 | 0.009 | 0.183 | 0.001 | 38.726 |

Abbreviation: SNP, single-nucleotide polymorphism; MR, Mendelian Randomisation; EAF, effect allele frequency; MAF, minor allele frequency; HDL, high-density lipoprotein; chr, chromosome.

**Supplemental Table 5. Instruments for Drug Target Genes (related to Supplemental Table 11).**

| **Target** | **SNP** | **Chromosome** | **Position** | **Effect allele** | **Other allele** | **Beta** | **Se** | ***P***-val | **Samplesize** |
| --- | --- | --- | --- | --- | --- | --- | --- | --- | --- |
| *PPARA* | rs11705132 | 22 | 46728858 | T | C | 0.145263 | 0.01927076 | 4.7797E-14 | 28870 |
| *PPARA* | rs117135869 | 22 | 46664412 | T | C | -0.238688 | 0.04241608 | 1.8295E-08 | 19400 |
| *PPARA* | rs129600 | 22 | 46557161 | G | A | -0.07850869 | 0.00929042 | 2.8981E-17 | 27185 |
| *DGAT2* | rs117111120 | 11 | 75434829 | T | C | -0.1104287 | 0.01936157 | 1.173E-08 | 30077 |
| *DGAT2* | rs1944438 | 11 | 75483623 | G | A | 0.05431752 | 0.008039774 | 1.4185E-11 | 30902 |
| *DGAT2* | rs575976 | 11 | 75437630 | G | A | -0.06751782 | 0.009930552 | 1.0535E-11 | 31684 |
| *DGAT2* | rs60677263 | 11 | 75513888 | G | A | -0.2565517 | 0.01315333 | 9.9947E-85 | 31569 |
| *ELOVL4* | rs10943669 | 6 | 80583474 | G | A | -0.083510641 | 0.007958548 | 9.2634E-26 | 31684 |
| *ELOVL4* | rs116965271 | 6 | 80749841 | A | G | 0.1810459 | 0.02726143 | 3.1156E-11 | 27423 |
| *ELOVL4* | rs12207890 | 6 | 80670767 | G | A | 0.06250931 | 0.01076726 | 6.4213E-09 | 31355 |
| *ELOVL4* | rs13211239 | 6 | 80755314 | T | G | -0.2161364 | 0.03794264 | 1.2236E-08 | 21960 |
| *ELOVL4* | rs1321845 | 6 | 80575820 | T | C | 0.06252344 | 0.007990726 | 5.1008E-15 | 31678 |
| *ELOVL4* | rs191828662 | 6 | 80586182 | G | A | -0.1751557 | 0.03036786 | 8.0313E-09 | 25543 |
| *ELOVL4* | rs239511 | 6 | 80693816 | A | G | 0.2309911 | 0.007889094 | 1.8754E-188 | 31684 |
| *ELOVL4* | rs41270555 | 6 | 80625716 | T | C | 0.2116483 | 0.0258916 | 2.9784E-16 | 29241 |
| *ELOVL4* | rs62405996 | 6 | 80756377 | G | A | -0.1220442 | 0.01394679 | 2.1185E-18 | 29828 |
| *ELOVL4* | rs73747963 | 6 | 80718513 | A | G | -0.151033 | 0.01828554 | 1.4592E-16 | 31684 |
| *ELOVL4* | rs79363916 | 6 | 80712748 | C | T | 0.1744334 | 0.0289872 | 1.7724E-09 | 29216 |
| *LPL* | rs10106796 | 8 | 19735624 | C | T | 0.15022164 | 0.009969911 | 2.6535E-51 | 31684 |
| *LPL* | rs113831503 | 8 | 19825055 | T | C | -0.2049025 | 0.02311391 | 7.6504E-19 | 31179 |
| *LPL* | rs11774936 | 8 | 19723883 | A | G | 0.1168706 | 0.01545028 | 3.9026E-14 | 31355 |
| *LPL* | rs13252698 | 8 | 19923297 | T | C | -0.237356 | 0.008590329 | 4.7743E-168 | 31567 |
| *LPL* | rs141450316 | 8 | 19743062 | T | C | -0.2360952 | 0.03953104 | 2.335E-09 | 20854 |
| *LPL* | rs142084074 | 8 | 19768150 | A | G | 0.5041642 | 0.03789113 | 2.1476E-40 | 25544 |
| *LPL* | rs268 | 8 | 19813529 | G | A | -0.1916982 | 0.0304917 | 3.2366E-10 | 27408 |
| *LPL* | rs308 | 8 | 19817476 | G | T | 0.2397431 | 0.02734203 | 1.8155E-18 | 29953 |
| *LPL* | rs316 | 8 | 19818436 | A | C | -0.08907092 | 0.01189039 | 6.8351E-14 | 31684 |
| *LPL* | rs34266755 | 8 | 19900474 | T | C | -0.1226532 | 0.01282487 | 1.1351E-21 | 30077 |
| *LPL* | rs3779787 | 8 | 19797916 | T | G | 0.3506297 | 0.01093667 | 1E-200 | 31477 |
| *LPL* | rs3898938 | 8 | 19749390 | T | C | 0.1287981 | 0.007924623 | 2.1339E-59 | 31684 |
| *LPL* | rs4389957 | 8 | 19862746 | G | A | -0.1612904 | 0.00796535 | 3.6252E-91 | 31566 |
| *LPL* | rs6586870 | 8 | 19709775 | A | G | -0.09452157 | 0.008164459 | 5.3649E-31 | 31684 |
| *LPL* | rs6586878 | 8 | 19758457 | T | C | -0.1190147 | 0.008768619 | 5.7903E-42 | 31569 |
| *LPL* | rs7005359 | 8 | 19870693 | G | A | 0.3983793 | 0.01014765 | 1E-200 | 31355 |
| *LPL* | rs77352983 | 8 | 19720813 | T | C | -0.09955465 | 0.01805325 | 3.4957E-08 | 28673 |
| *LPL* | rs77842142 | 8 | 19902490 | T | C | 0.7547719 | 0.06659126 | 8.884E-30 | 8518 |
| *HMGCR* | rs148880478 | 5 | 74564351 | T | C | 0.1340787 | 0.02033683 | 4.3161E-11 | 28314 |
| *HMGCR* | rs59014134 | 5 | 74589385 | T | C | 0.05945977 | 0.009420862 | 2.7652E-10 | 31564 |
| *HMGCR* | rs6453133 | 5 | 74692776 | G | A | 0.1277631 | 0.008536488 | 1.2137E-50 | 31684 |
| *CETP* | rs1532624 | 16 | 57005479 | A | C | -0.1300508 | 0.008051587 | 1.0958E-58 | 31086 |
| *CETP* | rs1651666 | 16 | 57056508 | T | C | 0.1074929 | 0.008585351 | 5.7743E-36 | 31470 |
| *CETP* | rs1800776 | 16 | 56995234 | A | C | 0.2630669 | 0.01671741 | 8.5692E-56 | 31351 |
| *CETP* | rs1968905 | 16 | 57010948 | T | G | -0.08583592 | 0.01023696 | 5.0726E-17 | 31340 |
| *CETP* | rs289726 | 16 | 57074451 | T | C | -0.05452144 | 0.008252946 | 3.9378E-11 | 31470 |
| *CETP* | rs4783966 | 16 | 57068360 | A | G | -0.05833036 | 0.009479371 | 7.5839E-10 | 31470 |

Abbreviation: SNP, single-nucleotide polymorphism; *PPARA*, Peroxisome proliferator-activated receptor alpha; *DGAT2*, Diacylglycerol O-acyltransferase 2; *ELOVL4*, Elongation of very long chain fatty acids protein 4; *LPL*, Lipoprotein lipase; *HMGCR*, 3-Hydroxy-3-Methylglutaryl-CoA Reductase; *CETP*, Cholesteryl Ester Transfer Protein.

**Supplemental Table 6. Correlation analysis between triglycerides and HDL Subcomponents.**

|  | **Total Triglycerides** |
| --- | --- |
| HDL Cholesterol | -0.410^***^ |
| Triglycerides in HDL | 0.835^***^ |
| Phospholipids in HDL | -0.164^***^ |
| Cholesteryl Esters in HDL | -0.449^***^ |
| Free Cholesterol in HDL | -0.254^***^ |
| Total Lipids in HDL | -0.226^***^ |
| Concentration of HDL Particles | -0.074^***^ |
| Average Diameter for HDL Particles | -0.486^***^ |
| Concentration of VLDL Particles | 0.844^***^ |
| Triglycerides in VLDL | 0.992^***^ |
| VLDL Cholesterol | 0.763^***^ |
| Concentration of LDL Particles | 0.485^***^ |
| Triglycerides in LDL | 0.829^***^ |
| LDL Cholesterol | 0.351^***^ |

Abbreviation: HDL, high density lipoprotein; VLDL, very low-density lipoprotein; LDL, low density lipoprotein.

Correlations were estimated using the Spearman correlation method.

^***^ *P*<0.001

**Supplemental Table 7. Association between HDL-P and Incident Coronary Artery Disease Events across Triglyceride Levels.**

|  | **Events/N** | **Crude model** | | **Model 1** | | **Model 2** | |
| --- | --- | --- | --- | --- | --- | --- | --- |
|  |  | **HR (95% CI)** | ***P*_value** | **HR (95% CI)** | ***P*_value** | **HR (95% CI)** | ***P*_value** |
| **Low-normal triglycerides** | | | | | | | |
| HDL-P Tertile1 | 1830/26295 | 1 [Ref] |  | 1 [Ref] |  | 1 [Ref] |  |
| HDL-P Tertile2 | 1298/26281 | 0.695(0.647,0.746) | <0.001 | 0.786(0.732,0.845) | <0.001 | 0.847(0.787,0.911) | <0.001 |
| HDL-P Tertile3 | 1249/26282 | 0.671(0.624,0.721) | <0.001 | 0.796(0.738,0.859) | <0.001 | 0.847(0.783,0.916) | <0.001 |
| **High-normal triglycerides** | | | | | | | |
| HDL-P Tertile1 | 2533/25214 | 1 [Ref] |  | 1 [Ref] |  | 1 [Ref] |  |
| HDL-P Tertile2 | 1914/25198 | 0.739(0.696,0.784) | <0.001 | 0.804(0.757,0.854) | <0.001 | 0.884(0.831,0.939) | <0.001 |
| HDL-P Tertile3 | 1579/25200 | 0.606(0.569,0.646) | <0.001 | 0.703(0.657,0.751) | <0.001 | 0.788(0.735,0.844) | <0.001 |
| **Borderline hypertriglyceridemia** | | | | | | | |
| HDL-P Tertile1 | 2489/21944 | 1 [Ref] |  | 1 [Ref] |  | 1 [Ref] |  |
| HDL-P Tertile2 | 1360/15131 | 0.775(0.726,0.828) | <0.001 | 0.835(0.781,0.894) | <0.001 | 0.888(0.829,0.951) | <0.001 |
| HDL-P Tertile3 | 806/10618 | 0.652(0.602,0.705) | <0.001 | 0.742(0.682,0.806) | <0.001 | 0.791(0.726,0.862) | <0.001 |
| **Moderate hypertriglyceridemia** | | | | | | | |
| HDL-P Tertile1 | 3588/26975 | 1 [Ref] |  | 1 [Ref] |  | 1 [Ref] |  |
| HDL-P Tertile2 | 1886/17347 | 0.800(0.756,0.846) | <0.001 | 0.842(0.796,0.891) | <0.001 | 0.869(0.820,0.921) | <0.001 |
| HDL-P Tertile3 | 1013/10462 | 0.709(0.661,0.760) | <0.001 | 0.784(0.729,0.842) | <0.001 | 0.797(0.740,0.859) | <0.001 |
| **Severe hypertriglyceridemia** | | | | | | | |
| HDL-P Tertile1 | 170/1065 | 1 [Ref] |  | 1 [Ref] |  | 1 [Ref] |  |
| HDL-P Tertile2 | 84/731 | 0.687(0.529,0.892) | 0.005 | 0.714(0.548,0.929) | 0.012 | 0.768(0.584,1.010) | 0.059 |
| HDL-P Tertile3 | 55/421 | 0.779(0.575,1.056) | 0.108 | 0.820(0.600,1.121) | 0.213 | 0.846(0.604,1.185) | 0.332 |

Crude model was crude analysis without adjustment.

Model 1 adjusted for age, sex, and ethnic background.

Model 2 was adjusted as in Model 1 and for overall health rating, education qualifications, smoking status, alcohol drinker status, assessment centre, Townsend deprivation index, history of diabetes, history of hypertension, lipid-lowering therapy, antihypertensive therapy, insulin therapy, systolic blood pressure, glucose, body mass index, and total triglyceride.

Abbreviation: HDL, high density lipoprotein; HDL-P, Concentration of HDL Particles;HR, hazard ratio; CI, confidence intervals.

**Supplemental Table 8. Association between HDL-C and Incident Coronary Artery Disease Events across Triglyceride Levels.**

|  | **Events/N** | **Crude model** | | **Model 1** | | **Model 2** | |
| --- | --- | --- | --- | --- | --- | --- | --- |
|  |  | **HR (95% CI)** | ***P*_value** | **HR (95% CI)** | ***P*_value** | **HR (95% CI)** | ***P*_value** |
| **Low-normal triglycerides** |  |  |  |  |  |  |  |
| HDL-C Tertile1 | 1946/26288 | 1 [Ref] |  | 1 [Ref] |  | 1 [Ref] |  |
| HDL-C Tertile2 | 1301/26287 | 0.656(0.612,0.704) | <0.001 | 0.771(0.717,0.829) | <0.001 | 0.865(0.804,0.932) | <0.001 |
| HDL-C Tertile3 | 1130/26283 | 0.570(0.529,0.613) | <0.001 | 0.708(0.654,0.767) | <0.001 | 0.813(0.749,0.884) | <0.001 |
| **High-normal triglycerides** |  |  |  |  |  |  |  |
| HDL-C Tertile1 | 2653/25204 | 1 [Ref] |  | 1 [Ref] |  | 1 [Ref] |  |
| HDL-C Tertile2 | 1897/25207 | 0.699(0.659,0.741) | <0.001 | 0.755(0.711,0.802) | <0.001 | 0.848(0.797,0.902) | <0.001 |
| HDL-C Tertile3 | 1476/25201 | 0.540(0.507,0.576) | <0.001 | 0.627(0.585,0.672) | <0.001 | 0.759(0.706,0.816) | <0.001 |
| **Borderline hypertriglyceridemia** |  |  |  |  |  |  |  |
| HDL-C Tertile1 | 3618/33959 | 1 [Ref] |  | 1 [Ref] |  | 1 [Ref] |  |
| HDL-C Tertile2 | 825/10182 | 0.750(0.695,0.809) | <0.001 | 0.820(0.759,0.887) | <0.001 | 0.901(0.833,0.975) | 0.01 |
| HDL-C Tertile3 | 212/3553 | 0.548(0.477,0.629) | <0.001 | 0.636(0.552,0.733) | <0.001 | 0.712(0.618,0.822) | <0.001 |
| **Moderate hypertriglyceridemia** |  |  |  |  |  |  |  |
| HDL-C Tertile1 | 5639/45737 | 1 [Ref] |  | 1 [Ref] |  | 1 [Ref] |  |
| HDL-C Tertile2 | 697/7301 | 0.761(0.704,0.824) | <0.001 | 0.833(0.769,0.903) | <0.001 | 0.898(0.828,0.973) | 0.009 |
| HDL-C Tertile3 | 151/1743 | 0.693(0.590,0.815) | <0.001 | 0.794(0.675,0.935) | 0.006 | 0.873(0.741,1.029) | 0.106 |
| **Severe hypertriglyceridemia** |  |  |  |  |  |  |  |
| HDL-C Tertile1 | 280/2004 | 1 [Ref] |  | 1 [Ref] |  | 1 [Ref] |  |
| HDL-C Tertile2 | 24/186 | 0.917(0.604,1.391) | 0.682 | 0.99(0.651,1.508) | 0.964 | 1.017(0.665,1.557) | 0.938 |
| HDL-C Tertile3 | 5/27 | 1.380(0.570,3.341) | 0.476 | 1.641(0.672,4.008) | 0.277 | 1.858(0.753,4.584) | 0.179 |

Crude model was crude analysis without adjustment.

Model 1 adjusted for age, sex, and ethnic background.

Model 2 was adjusted as in Model 1 and for overall health rating, education qualifications, smoking status, alcohol drinker status, assessment centre, Townsend deprivation index, history of diabetes, history of hypertension, lipid-lowering therapy, antihypertensive therapy, insulin therapy, systolic blood pressure, glucose, body mass index, and total triglyceride.

Abbreviation: HDL, high density lipoprotein; HDL-C, HDL Cholesterol; HR, hazard ratio; CI, confidence intervals.

**Supplemental Table 9. Association of Triglycerides in HDL with Incident CAD Events by Percentile Ranges.**

| **Triglycerides in HDL** | **Events/N** | **Crude model** | | **Model 1** | | **Model 2** | |
| --- | --- | --- | --- | --- | --- | --- | --- |
|  |  | **HR (95% CI)** | ***P*_value** | **HR (95% CI)** | ***P*_value** | **HR (95% CI)** | ***P*_value** |
| Continuous | 21854/259166 | 1.190(1.176,1.205) | <0.001 | 1.145(1.131,1.159) | <0.001 | 1.066(1.052,1.080) | <0.001 |
| 1th - 50th percentile | 9257/129595 | ref |  | ref |  | ref |  |
| 51th - 95th percentile | 11130/116612 | 1.360(1.323,1.398) | <0.001 | 1.229(1.195,1.264) | <0.001 | 1.109(1.078,1.140) | <0.001 |
| 96th - 100th percentile | 1467/12959 | 1.636(1.549,1.729) | <0.001 | 1.509(1.428,1.595) | <0.001 | 1.197(1.132,1.266) | <0.001 |

Crude model was crude analysis without adjustment.

Model 1 adjusted for age, sex, and ethnic background.

Model 2 was adjusted as in Model 1 and for overall health rating, education qualifications, smoking status, alcohol drinker status, assessment centre, Townsend deprivation index, history of diabetes, history of hypertension, lipid-lowering therapy, antihypertensive therapy, insulin therapy, systolic blood pressure, glucose, and body mass index.

Abbreviation: CAD, coronary artery disease; HDL, high density lipoprotein; HR, hazard ratio; CI, confidence intervals.

**Supplemental Table 10. Causal effects of Triglycerides in HDL on CAD using different methods.**

| **Group** | **Exposure** | **Inverse variance weighted** | | **Weighted median** | | **MR Egger** | | **Weighted mode** | | **Pleiotropy test by MR Egger** | | **Cochran's Q test** | | **MR-Presso** | | | | | |
| --- | --- | --- | --- | --- | --- | --- | --- | --- | --- | --- | --- | --- | --- | --- | --- | --- | --- | --- | --- |
|  |  |  |  |  |  |  |  |  |  |  |  |  |  | **Global Test** | | **Outlier-corrected inverse variance weighted** | | **Distortion Test** | |
|  |  | **OR (95% CI)** | ***P* value** | **OR (95% CI)** | ***P* value** | **OR (95% CI)** | ***P* value** | **OR (95% CI)** | ***P* value** | **Intercept (SE)** | ***P* value** | **Q (df)** | ***P* value** | **RSS** | ***P* value** | **OR (95% CI)** | ***P* value** | **Coefficient** | ***P* value** |
| Dataset group 1 | HDL-TG | 1.12(1.053,1.192) | <0.001 | 1.156(1.086,1.231) | <0.001 | 1.009(0.91,1.12) | 0.862 | 1.152(1.089,1.219) | <0.001 | 0.008(0.003) | 0.016 | 970.691(232) | <0.001 | 996.359 | <0.001 | 1.18(1.128,1.235) | <0.001 | -26.220 | 0.170 |
| Dataset group 2 | HDL-TG | 1.141(1.032,1.263) | 0.010 | 1.144(1.045,1.253) | 0.003 | 1.156(0.946,1.412) | 0.176 | 1.147(1.051,1.253) | 0.007 | -0.001(0.01) | 0.884 | 36.812(16) | 0.002 | - | | | | | |

Abbreviation: HDL, high density lipoprotein; HDL-TG, Triglycerides in HDL; CAD, coronary artery disease; OR, odds ratio; CI, confidence intervals.

**Supplement Table 11. Mendelian Randomization Associations of Known Targets of Lipid-lowering Genetic Variants in Drug Target Gene Loci with the Risk of HDL-TG Elevation.**

| **Treatment proxy** | **Exposure** | **Outcome** | **Inverse variance weighted** | | **Weighted median** | | **MR Egger** | | **Weighted mode** | | **Pleiotropy test by MR Egger** | | **Cochran's Q test** | |
| --- | --- | --- | --- | --- | --- | --- | --- | --- | --- | --- | --- | --- | --- | --- |
|  |  |  | **β (SE)** | ***P-*value** | **β (SE)** | ***P*-value** | **β (SE)** | ***P*-value** | **β (SE)** | ***P*-value** | **Intercept (SE)** | ***P*-value** | **Q (df)** | ***P*-value** |
| Fenofibric acid | *PPARA* | HDL-TG | 0.082  (0.038) | 0.030 | 0.074  (0.045) | 0.098 | 0.079  (0.122) | 0.634 | 0.065  (0.054) | 0.355 | 0  (0.013) | 0.984 | 1.522(2) | 0.467 |
| omega-3 fatty acids | *DGAT2* | HDL-TG | 0.106  (0.024) | <0.001 | 0.111  (0.027) | <0.001 | 0.134  (0.040) | 0.080 | 0.118  (0.028) | 0.024 | -0.004  (0.005) | 0.479 | 1.322(3) | 0.724 |
|  | *ELOVL4* | HDL-TG | -0.048  (0.019) | 0.010 | -0.026  (0.017) | 0.135 | -0.011  (0.038) | 0.789 | -0.021  (0.017) | 0.249 | -0.006  (0.005) | 0.288 | 18.905(10) | 0.041 |
|  | *LPL* | HDL-TG | -0.175  (0.031) | <0.001 | -0.189  (0.011) | <0.001 | -0.216  (0.065) | 0.004 | -0.195  (0.013) | <0.001 | 0.010  (0.013) | 0.479 | 332.743(17) | <0.001 |
| Statins | *HMGCR* | HDL-TG | 0.013  (0.030) | 0.654 | 0.010  (0.031) | 0.749 | -0.016  (0.098) | 0.896 | -0.003  (0.034) | 0.934 | 0.003  (0.011) | 0.805 | 1.22(2) | 0.543 |
| - | *CETP* | HDL-TG | 0.143(0.068) | 0.036 | 0.076(0.026) | 0.003 | 0.155(0.166) | 0.403 | 0.051(0.026) | 0.111 | -0.001(0.019) | 0.94 | 78.385(5) | <0.001 |

Abbreviation: HDL-TG, Triglycerides in HDL; *PPARA*, Peroxisome proliferator-activated receptor alpha; *DGAT2*, Diacylglycerol O-acyltransferase 2; *ELOVL4*, Elongation of very long chain fatty acids protein 4; *LPL*, Lipoprotein lipase; *HMGCR*, 3-Hydroxy-3-Methylglutaryl-CoA Reductase; *CETP*, Cholesteryl Ester Transfer Protein; SE, standard errors.

**Supplemental Table 12. Baseline Characteristics of VLDL Subcomponents by Grouping of Triglyceride Levels.**

| **VLDL subcomponents (median [IQR])** | **All population** | **Low-normal triglycerides** | **High-normal triglycerides** | **Borderline hypertriglyceridemia** | **Moderate hypertriglyceridemia** | **Severe hypertriglyceridemia** | ***P* overall** | ***P* trend** |
| --- | --- | --- | --- | --- | --- | --- | --- | --- |
|  | **N=259166** | **N=78858** | **N=75612** | **N=47694** | **N=54785** | **N=2217** |  |  |
| Average Diameter for VLDL Particles (nm) | 38.6 [37.7;39.5] | 37.4 [36.9;37.9] | 38.4 [38.0;38.9] | 39.3 [38.8;39.7] | 40.0 [39.5;40.5] | 40.9 [40.2;41.5] | <0.001 | <0.001 |
| Concentration of VLDL Particles (mmol/L) | 0.00 [0.00;0.00] | 0.00 [0.00;0.00] | 0.00 [0.00;0.00] | 0.00 [0.00;0.00] | 0.00 [0.00;0.00] | 0.00 [0.00;0.00] | <0.001 | <0.001 |
| Total Lipids in VLDL (mmol/L) | 2.00 [1.48;2.65] | 1.29 [1.06;1.54] | 1.94 [1.68;2.21] | 2.50 [2.17;2.82] | 3.13 [2.67;3.63] | 3.86 [3.10;4.80] | <0.001 | <0.001 |
| VLDL Cholesterol (mmol/L) | 0.71 [0.55;0.88] | 0.52 [0.42;0.63] | 0.71 [0.59;0.83] | 0.82 [0.68;0.97] | 0.91 [0.75;1.08] | 0.92 [0.73;1.14] | <0.001 | <0.001 |
| Triglycerides in VLDL (mmol/L) | 0.83 [0.55;1.20] | 0.45 [0.36;0.55] | 0.78 [0.67;0.90] | 1.11 [0.97;1.25] | 1.53 [1.30;1.79] | 2.16 [1.68;2.73] | <0.001 | <0.001 |
| Phospholipids in VLDL (mmol/L) | 0.46 [0.34;0.59] | 0.31 [0.25;0.37] | 0.44 [0.38;0.51] | 0.55 [0.47;0.63] | 0.67 [0.57;0.78] | 0.79 [0.63;0.97] | <0.001 | <0.001 |
| Cholesteryl Esters in VLDL (mmol/L) | 0.42 [0.33;0.52] | 0.33 [0.27;0.40] | 0.43 [0.35;0.51] | 0.49 [0.40;0.58] | 0.52 [0.42;0.62] | 0.48 [0.39;0.60] | <0.001 | <0.001 |
| Free Cholesterol in VLDL (mmol/L) | 0.28 [0.21;0.36] | 0.20 [0.16;0.24] | 0.28 [0.23;0.32] | 0.34 [0.29;0.39] | 0.40 [0.33;0.47] | 0.44 [0.35;0.54] | <0.001 | <0.001 |

Abbreviation: VLDL, very low-density lipoprotein; IQR, interquartile range.

**Supplemental Table 13. Baseline Characteristics of LDL Subcomponents by Grouping of Triglyceride Levels.**

| **LDL subcomponents (median [IQR])** | **All population** | **Low-normal triglycerides** | **High-normal triglycerides** | **Borderline hypertriglyceridemia** | **Moderate hypertriglyceridemia** | **Severe hypertriglyceridemia** | ***P* overall** | ***P* trend** |
| --- | --- | --- | --- | --- | --- | --- | --- | --- |
|  | **N=259166** | **N=78858** | **N=75612** | **N=47694** | **N=54785** | **N=2217** |  |  |
| Average Diameter for LDL Particles (nm) | 23.9 [23.9;24.0] | 24.0 [23.9;24.0] | 23.9 [23.9;24.0] | 23.9 [23.9;24.0] | 23.9 [23.8;23.9] | 23.8 [23.7;23.9] | <0.001 | <0.001 |
| Concentration of LDL Particles (mmol/L) | 0.00 [0.00;0.00] | 0.00 [0.00;0.00] | 0.00 [0.00;0.00] | 0.00 [0.00;0.00] | 0.00 [0.00;0.00] | 0.00 [0.00;0.00] | <0.001 | <0.001 |
| Total Lipids in LDL (mmol/L) | 2.49 [2.12;2.90] | 2.30 [1.97;2.65] | 2.52 [2.16;2.89] | 2.63 [2.23;3.04] | 2.68 [2.26;3.13] | 2.39 [1.93;2.88] | <0.001 | <0.001 |
| LDL Cholesterol (mmol/L) | 1.74 [1.47;2.04] | 1.62 [1.38;1.88] | 1.77 [1.50;2.04] | 1.83 [1.55;2.14] | 1.84 [1.54;2.17] | 1.56 [1.23;1.93] | <0.001 | <0.001 |
| Triglycerides in LDL (mmol/L) | 0.14 [0.12;0.17] | 0.11 [0.10;0.13] | 0.14 [0.12;0.15] | 0.15 [0.14;0.17] | 0.19 [0.17;0.22] | 0.26 [0.22;0.31] | <0.001 | <0.001 |
| Phospholipids in LDL (mmol/L) | 0.61 [0.52;0.70] | 0.57 [0.49;0.65] | 0.62 [0.53;0.71] | 0.64 [0.54;0.74] | 0.64 [0.54;0.75] | 0.56 [0.45;0.67] | <0.001 | <0.001 |
| Cholesteryl Esters in LDL (mmol/L) | 1.28 [1.07;1.50] | 1.16 [0.99;1.35] | 1.29 [1.10;1.49] | 1.35 [1.14;1.57] | 1.39 [1.16;1.63] | 1.23 [0.98;1.50] | <0.001 | <0.001 |
| Free Cholesterol in LDL (mmol/L) | 0.47 [0.39;0.55] | 0.46 [0.39;0.53] | 0.48 [0.40;0.56] | 0.48 [0.40;0.56] | 0.46 [0.37;0.54] | 0.34 [0.25;0.43] | <0.001 | 0.043 |

Abbreviation: LDL, low density lipoprotein; IQR, interquartile range.

**Supplemental Table 14. Association of Subcomponents in VLDL with Incident Coronary Artery Disease Events.**

|  | **Crude** | | **Model 1** | | **Model 2** | |
| --- | --- | --- | --- | --- | --- | --- |
|  | **HR (95% CI)** | ***P*_value** | **HR (95% CI)** | ***P*_value** | **HR (95% CI)** | ***P*_value** |
| Average Diameter for VLDL Particles | 1.293(1.276,1.31) | <0.001 | 1.166(1.15,1.183) | <0.001 | 1.046(1.031,1.062) | <0.001 |
| Concentration of VLDL Particles | 1.186(1.171,1.201) | <0.001 | 1.145(1.131,1.16) | <0.001 | 1.162(1.147,1.178) | <0.001 |
| Total Lipids in VLDL | 1.23(1.215,1.245) | <0.001 | 1.165(1.151,1.18) | <0.001 | 1.126(1.111,1.14) | <0.001 |
| VLDL Cholesterol | 1.139(1.124,1.154) | <0.001 | 1.115(1.1,1.13) | <0.001 | 1.169(1.153,1.185) | <0.001 |
| Triglycerides in VLDL | 1.25(1.236,1.264) | <0.001 | 1.171(1.156,1.185) | <0.001 | 1.09(1.076,1.104) | <0.001 |
| Phospholipids in VLDL | 1.216(1.201,1.231) | <0.001 | 1.162(1.148,1.177) | <0.001 | 1.147(1.132,1.162) | <0.001 |
| Cholesteryl Esters in VLDL | 1.098(1.083,1.112) | <0.001 | 1.091(1.077,1.105) | <0.001 | 1.175(1.159,1.192) | <0.001 |
| Free Cholesterol in VLDL | 1.188(1.173,1.203) | <0.001 | 1.143(1.128,1.158) | <0.001 | 1.153(1.138,1.169) | <0.001 |

Model 1 adjusted for age, sex, and ethnic background.

Model 2 was adjusted as in Model 1 and for overall health rating, education qualifications, smoking status, alcohol drinker status, assessment centre, Townsend deprivation index, history of diabetes, history of hypertension, lipid-lowering therapy, antihypertensive therapy, insulin therapy, systolic blood pressure, glucose, and body mass index.

Abbreviation: VLDL, very low-density lipoprotein; HR, hazard ratio; CI, confidence intervals.

**Supplemental Table 15. Association of Subcomponents in LDL with Incident Coronary Artery Disease Events.**

|  | **Crude** | | **Model 1** | | **Model 2** | |
| --- | --- | --- | --- | --- | --- | --- |
|  | **HR (95% CI)** | ***P*_value** | **HR (95% CI)** | ***P*_value** | **HR (95% CI)** | ***P*_value** |
| Average Diameter for LDL Particles | 0.843(0.832,0.853) | <0.001 | 0.931(0.919,0.944) | <0.001 | 1.025(1.011,1.039) | <0.001 |
| Concentration of LDL Particles | 1.041(1.028,1.055) | <0.001 | 1.049(1.036,1.063) | <0.001 | 1.161(1.145,1.177) | <0.001 |
| Total Lipids in LDL | 0.995(0.982,1.009) | 0.486 | 1.017(1.003,1.03) | 0.015 | 1.126(1.11,1.142) | <0.001 |
| LDL Cholesterol | 0.972(0.959,0.985) | <0.001 | 0.999(0.986,1.013) | 0.915 | 1.117(1.102,1.133) | <0.001 |
| Triglycerides in LDL | 1.242(1.228,1.256) | <0.001 | 1.193(1.179,1.207) | <0.001 | 1.128(1.114,1.142) | <0.001 |
| Phospholipids in LDL | 0.992(0.978,1.005) | 0.214 | 1.014(1.001,1.028) | 0.036 | 1.128(1.112,1.144) | <0.001 |
| Cholesteryl Esters in LDL | 0.997(0.984,1.01) | 0.653 | 1.016(1.002,1.029) | 0.021 | 1.122(1.106,1.137) | <0.001 |
| Free Cholesterol in LDL | 0.907(0.895,0.92) | <0.001 | 0.956(0.943,0.969) | <0.001 | 1.099(1.083,1.114) | <0.001 |

Model 1 adjusted for age, sex, and ethnic background.

Model 2 was adjusted as in Model 1 and for overall health rating, education qualifications, smoking status, alcohol drinker status, assessment centre, Townsend deprivation index, history of diabetes, history of hypertension, lipid-lowering therapy, antihypertensive therapy, insulin therapy, systolic blood pressure, glucose, and body mass index.

Abbreviation: LDL, low density lipoprotein; HR, hazard ratio; CI, confidence intervals.

**Supplemental Table 16. Causal effects of Triglycerides in VLDL on CAD using different methods.**

| **Group** | **Exposure** | **Inverse variance weighted** | | **Weighted median** | | **MR Egger** | | **Weighted mode** | | **Pleiotropy test by MR Egger** | | **Cochran's Q test** | |
| --- | --- | --- | --- | --- | --- | --- | --- | --- | --- | --- | --- | --- | --- |
|  |  |  |  |  |  |  |  |  |  |  |  |  |  |
|  |  | **OR (95% CI)** | ***P* value** | **OR (95% CI)** | ***P* value** | **OR (95% CI)** | ***P* value** | **OR (95% CI)** | ***P* value** | **Intercept (SE)** | ***P* value** | **Q (df)** | ***P* value** |
| Dataset group 1 | VLDL-TG | 1.052(0.974,1.137) | 0.198 | 1.15(1.066,1.24) | <0.001 | 0.903(0.792,1.029) | 0.126 | 1.146(1.033,1.271) | 0.011 | 0.01(0.003) | 0.005 | 1230.465(238) | <0.001 |
| Dataset group 2 | VLDL-TG | 1.153(0.997,1.333) | 0.055 | 1.181(1.024,1.363) | 0.022 | 1.181(0.8,1.744) | 0.415 | 1.379(1.028,1.85) | 0.046 | -0.002(0.016) | 0.897 | 62.357(18) | <0.001 |

Abbreviation: VLDL, very low-density lipoprotein; VLDL-TG, Triglycerides in VLDL; CAD, coronary artery disease; MR, Mendelian Randomization; OR, odds ratio; CI, confidence intervals.

**Supplemental Table 17. Causal effects of Triglycerides in LDL on CAD using different methods.**

| **Group** | **Exposure** | **Inverse variance weighted** | | **Weighted median** | | **MR Egger** | | **Weighted mode** | | **Pleiotropy test by MR Egger** | | **Cochran's Q test** | | **MR-Presso** | | | | | |
| --- | --- | --- | --- | --- | --- | --- | --- | --- | --- | --- | --- | --- | --- | --- | --- | --- | --- | --- | --- |
|  |  |  |  |  |  |  |  |  |  |  |  |  |  | **Global Test** | | **Outlier-corrected inverse variance weighted** | | **Distortion Test** | |
|  |  | **OR (95% CI)** | ***P* value** | **OR (95% CI)** | ***P* value** | **OR (95% CI)** | ***P* value** | **OR (95% CI)** | ***P* value** | **Intercept (SE)** | ***P* value** | **Q (df)** | ***P* value** | **RSS** | ***P* value** | **OR (95% CI)** | ***P* value** | **Coefficient** | ***P* value** |
| Dataset group 1 | LDL-TG | 1.257(1.178,1.341) | <0.001 | 1.197(1.116,1.284) | <0.001 | 1.105(0.989,1.234) | 0.080 | 1.177(1.107,1.252) | <0.001 | 0.009(0.003) | 0.006 | 996.849(231) | <0.001 | 1018.218 | <0.003 | 1.296(1.241,1.352) | <0.001 | -12.75 | 0.143 |
| Dataset group 2 | LDL-TG | 1.215(1.071,1.378) | 0.002 | 1.151(1.053,1.258) | 0.002 | 1.069(0.836,1.367) | 0.602 | 1.146(1.051,1.249) | 0.006 | 0.014(0.012) | 0.253 | 78.247(19) | <0.001 | - | | | | | |

Abbreviation: LDL, low density lipoprotein; LDL-TG, Triglycerides in LDL; CAD, coronary artery disease; MR, Mendelian Randomization; OR, odds ratio; CI, confidence intervals.

**Supplemental Table 18. Causal effects of Triglycerides in LDL on CAD using multivariate mendelian randomization analysis method.**

| **Exposure** | **Outcome** | **Dataset group 1** | | **Dataset group 2** | |
| --- | --- | --- | --- | --- | --- |
|  |  | **OR (95% CI)** | *P* value | **OR (95% CI)** | *P* value |
| LDL_TG | CAD | 1.512(1.364,1.677) | <0.001 | 1.385(1.092,1.758) | 0.007 |

Multivariate: LDL_C, HDL_C, Total_TG

Abbreviation: LDL, low density lipoprotein; LDL-TG, Triglycerides in LDL; CAD, coronary artery disease; OR, odds ratio; CI, confidence intervals.

**Supplemental Table 19. Association of HDL-TG/HDL-C ratio with Incident Coronary Artery Disease Events.**

|  | **Crude** | | **Model 1** | | **Model 2** | |
| --- | --- | --- | --- | --- | --- | --- |
|  | **HR (95% CI)** | ***P*_value** | **HR (95% CI)** | ***P*_value** | **HR (95% CI)** | ***P*_value** |
| HDL-TG/HDL-C | 1.207(1.201,1.214) | <0.001 | 1.193(1.183,1.203) | <0.001 | 1.108(1.095,1.121) | <0.001 |

Model 1 adjusted for age, sex, and ethnic background.

Model 2 was adjusted as in Model 1 and for overall health rating, education qualifications, smoking status, alcohol drinker status, assessment centre, Townsend deprivation index, history of diabetes, history of hypertension, lipid-lowering therapy, antihypertensive therapy, insulin therapy, systolic blood pressure, glucose, and body mass index.

Abbreviation: HR, hazard ratio; CI, confidence intervals; HDL-TG, Triglycerides in high density lipoprotein; HDL-C, high density lipoprotein cholesterol.

**Supplemental Table 20. Comparison of Model Performance Metrics Between HDL-TG and HDL-TG/HDL-C Models for Predicting CAD Risk.**

|  | **Model with HDL-TG** | **Model with HDL-TG/HDL-C** |
| --- | --- | --- |
| Akaike Information Criterion (AIC) | 524879.3 | 524728.2 |
| Bayesian Information Criterion (BIC) | 525023.2 | 524872.1 |
| C-index | 0.7277 | 0.7292 |

Abbreviation: HDL-TG, Triglycerides in high density lipoprotein; HDL-C, high density lipoprotein cholesterol; CAD, coronary artery disease.

**Supplemental Table 21. Causal effects of HDL-C on CAD using different methods.**

| **Group** | **Exposure** | **Inverse variance weighted** | | **Weighted median** | | **MR Egger** | | **Weighted mode** | | **Pleiotropy test by MR Egger** | | **Cochran's Q test** | |
| --- | --- | --- | --- | --- | --- | --- | --- | --- | --- | --- | --- | --- | --- |
|  |  |  |  |  |  |  |  |  |  |  |  |  |  |
|  |  | **OR (95% CI)** | ***P* value** | **OR (95% CI)** | ***P* value** | **OR (95% CI)** | ***P* value** | **OR (95% CI)** | ***P* value** | **Intercept (SE)** | ***P* value** | **Q (df)** | ***P* value** |
| Dataset group 1 | HDL-C | 0.883(0.839,0.928) | <0.001 | 0.901(0.841,0.966) | 0.003 | 0.94(0.86,1.027) | 0.17 | 0.885(0.825,0.95) | 0.001 | -0.004(0.002) | 0.091 | 642.617(263) | <0.001 |
| Dataset group 2 | HDL-C | 0.994(0.815,1.214) | 0.956 | 1.137(0.956,1.352) | 0.147 | 1.203(0.64,2.261) | 0.576 | 1.276(1.031,1.58) | 0.043 | -0.014(0.022) | 0.543 | 52.092(13) | <0.001 |

Abbreviation: HDL-C, high density lipoprotein cholesterol; CAD, coronary artery disease; MR, Mendelian randomization; OR, odds ratio; CI, confidence intervals.

**Supplementary Figure**

**Supplemental Figure 1.** **Flow chart of participants.**


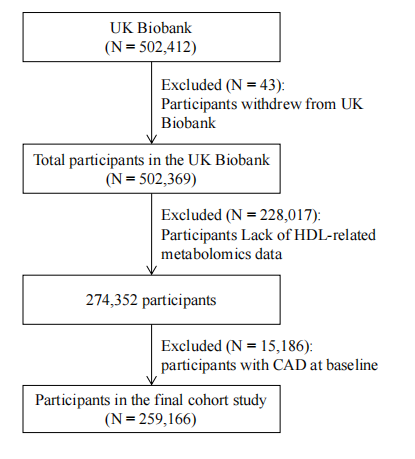


Abbreviation: HDL, high density lipoprotein; CAD, coronary artery disease.

**Supplemental Figure 2. Discordance Between HDL-TG and ApoB, LDL-C, and total-TG on Risk of CAD, as stratified by the 80th percentile.**


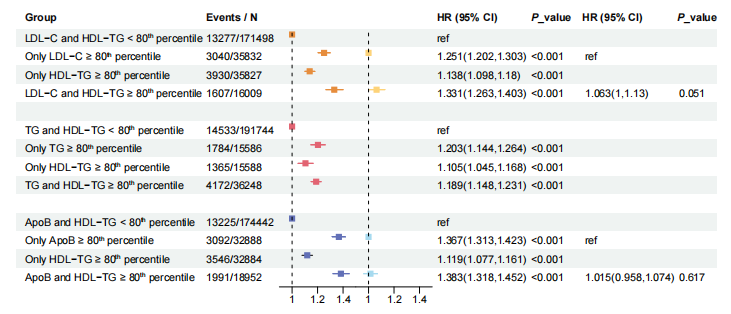


Model adjusted for age, sex, and ethnic background, overall health rating, education qualifications, smoking status, alcohol drinker status, assessment centre, Townsend deprivation index, history of diabetes, history of hypertension, lipid-lowering therapy, antihypertensive therapy, insulin therapy, systolic blood pressure, glucose, and body mass index.

Abbreviation: HDL-TG, Triglycerides in high density lipoprotein; ApoB, ApolipoproteinB; LDL-C, low-density lipoprotein cholesterol; TG, Triglycerides; CAD, coronary artery disease; HR, hazard ratio; CI, confidence intervals.

**Supplemental Figure 3. Association of Subcomponents in VLDL/LDL with Incident Coronary Artery Disease Events across Different Levels of VLDL/LDL Particle Diameter.**


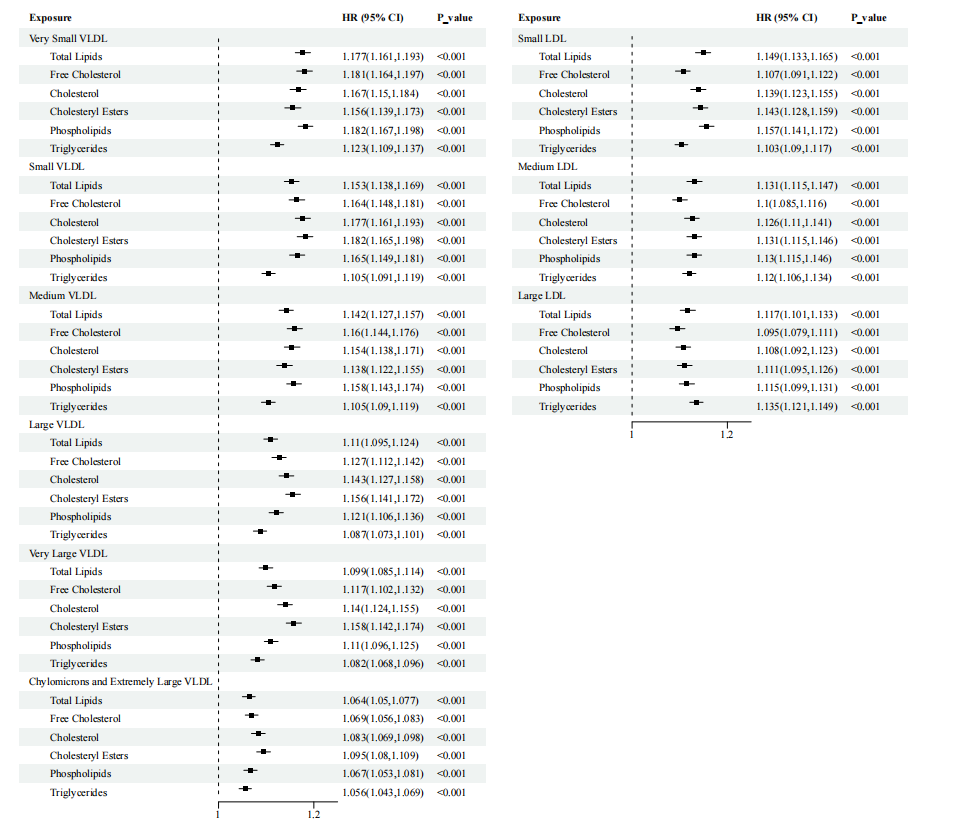


Model adjusted for age, sex, ethnic background, overall health rating, education qualifications, smoking status, alcohol drinker status, assessment centre, Townsend deprivation index, history of diabetes, history of hypertension, lipid-lowering therapy, antihypertensive therapy, insulin therapy, systolic blood pressure, glucose, and body mass index.

Abbreviation: VLDL, very low-density lipoprotein; LDL, low density lipoprotein; HR, hazard ratio; CI, confidence intervals.

**Supplemental Figure 4. Association between LDL-P/ LDL-C and Incident Coronary Artery Disease Events across Triglyceride Levels.**


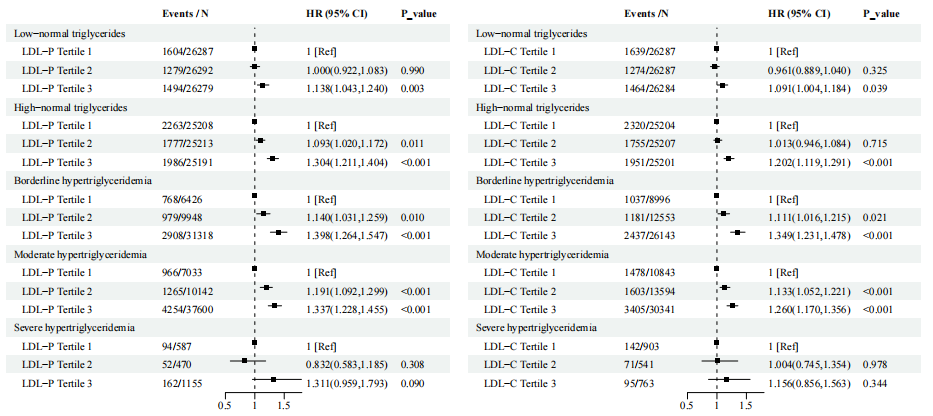


Model adjusted for age, sex, ethnic background, overall health rating, education qualifications, smoking status, alcohol drinker status, assessment centre, Townsend deprivation index, history of diabetes, history of hypertension, lipid-lowering therapy, antihypertensive therapy, insulin therapy, systolic blood pressure, glucose, body mass index, and total triglyceride.

Abbreviation: LDL, low density lipoprotein; LDL-P, Concentration of LDL Particles; LDL-C, LDL Cholesterol; HR, hazard ratio; CI, confidence intervals.
